# Adipose-Derived Stem Cells in Reinforced Collagen Gel: A Comparison between Two Approaches to Differentiation towards Smooth Muscle Cells

**DOI:** 10.3390/ijms24065692

**Published:** 2023-03-16

**Authors:** Elena Filova, Monika Supova, Adam Eckhardt, Marek Vrbacky, Andreu Blanquer, Martina Travnickova, Jarmila Knitlova, Tomas Suchy, Sarka Ryglova, Martin Braun, Zuzana Burdikova, Martin Schätz, Vera Jencova, Maxim Lisnenko, Lubos Behalek, Renata Prochazkova, Radek Sedlacek, Kristyna Kubasova, Lucie Bacakova

**Affiliations:** 1 Institute of Physiology of the Czech Academy of Sciences, Videnska 1083, 142 00 Prague 4, Czech Republic; 2 Department of Composites and Carbon Materials, the Institute of Rock Structure and Mechanics of the Czech Academy of Sciences, V Holesovickach 94/41, 182 09 Prague 8, Czech Republic; 3 Vinicna Microscopy Core Facility, Faculty of Science, Charles University, Albertov 6, 128 43 Prague 2, Czech Republic; 4 Department of Chemistry, Faculty of Science, Education and Humanities, Technical University of Liberec, Studentska 1402/2, 461 17 Liberec, Czech Republic; 5 Faculty of Mechanical Engineering, Technical University of Liberec, Studentska 1402/2, 461 17 Liberec, Czech Republic; 6 Regional Hospital Liberec, Husova 357/10, 460 63 Liberec, Czech Republic; 7 Faculty of Health Studies, Technical University of Liberec, Studentska 1402/2, 461 17 Liberec, Czech Republic; 8 Department of Mechanics, Biomechanics and Mechatronics, Faculty of Mechanical Engineering, Czech Technical University in Prague, Technicka 4, 166 00 Prague 6, Czech Republic

**Keywords:** collagen particles, stem cells differentiation, vascular patches, adipose tissue-derived stem cells, gel reinforcement, endothelial cells, extracellular matrix, remodelling, tissue engineering

## Abstract

Scaffolds made of degradable polymers, such as collagen, polyesters or polysaccharides, are promising matrices for fabrication of bioartificial vascular grafts or patches. In this study, collagen isolated from porcine skin was processed into a gel, reinforced with collagen particles and with incorporated adipose tissue-derived stem cells (ASCs). The cell-material constructs were then incubated in a DMEM medium with 2% of FS (DMEM_part), with added polyvinylalcohol nanofibers (PVA_part sample), and for ASCs differentiation towards smooth muscle cells (SMCs), the medium was supplemented either with human platelet lysate released from PVA nanofibers (PVA_PL_part) or with TGF-β1 + BMP-4 (TGF + BMP_part). The constructs were further endothelialised with human umbilical vein endothelial cells (ECs). The immunofluorescence staining of alpha-actin and calponin, and von Willebrand factor, was performed. The proteins involved in cell differentiation, the extracellular matrix (ECM) proteins, and ECM remodelling proteins were evaluated by mass spectrometry on day 12 of culture. Mechanical properties of the gels with ASCs were measured via an unconfined compression test on day 5. Gels evinced limited planar shrinkage, but it was higher in endothelialised TGF + BMP_part gel. Both PVA_PL_part samples and TGF + BMP_part samples supported ASC growth and differentiation towards SMCs, but only PVA_PL_part supported homogeneous endothelialisation. Young modulus of elasticity increased in all samples compared to day 0, and PVA_PL_part gel evinced a slightly higher ratio of elastic energy. The results suggest that PVA_PL_part collagen construct has the highest potential to remodel into a functional vascular wall.

## 1. Introduction

According to the World Health Organisation, cardiovascular diseases cause the deaths of 17.9 million people each year, which is 31% of all deaths worldwide [1]. The need for vascular prostheses and cardiovascular patches therefore increases every year. Autologous vascular grafts are used preferentially for the creation of small caliber prostheses, but they have limited availability [2]. For pulmonary artery reconstruction, the following materials have been used so far: branch patch homograft, bovine pericardium, autologous pericardium, and porcine intestinal submucosal patch [3]. Due to the increasing need for vascular grafts, scientists have been developing various tissue-engineered vascular prostheses from a wide range of polymers—either natural (e.g., collagen, gelatin, elastin, chitosan, cellulose, pullulan, decellularised tissues) or synthetic, such as poly(lactic acid) (PLA), poly(glycolic acid) (PGA), poly(caprolactone) (PCL), poly(vinyl alcohol) (PVA), dextran, chitosan, and their blends [4,5,6,7,8,9]. Collagen as a natural extracellular matrix polymer offers both mechanical support and binding sites for cell adhesion receptors. In addition, it can be prepared in the form of hydrogels, decellularised tissues, sponges, meshes, films, nano/micro-fibers, micro-particles, and sheets, or it can serve as a drug-delivery system [10,11,12,13,14]. For colonisation of these scaffolds, aortic smooth muscle cells (SMCs), endothelial cells (ECs), NIH3T3 fibroblasts, autologous endothelial progenitor cell (EPC)-derived ECs, and mesenchymal stem cells (MSCs) derived from the bone marrow or from the adipose tissue have been used [14,15,16]. Pure collagen hydrogels with entrapped cells have fast degradation, high contraction and weak mechanical properties. This unfavourable behavior of collagen gels could be overcome by preparing composites with other polymers, such as polydopamin, chitosan, hyaluronic acid, silk fibroin, or by reinforcement with, e.g., polycaprolactone fibers, coral collagen fibers or bioactive glass particles [17,18,19,20,21]. In addition, mechanical properties and stability of the collagen-based materials can be improved by crosslinking with various agents, e.g., epoxidised chitosan quaternary ammonium salt, using in situ photochemical crosslinking, genipin, 1-ethyl-3-(3-dimethylaminopropyl) carbodiimide hydrochloride (EDC)/N-hydroxysuccinimide (NHS) or transglutaminase [22,23,24,25,26]. However, the crosslinkers can be toxic and can be released from the material for a long time. 

Apart from biomechanical properties, it is important to improve bioactivity and bioconductivity of collagen gels. In various studies, the incorporation of vascular endothelial growth factor (VEGF), platelet-derived growth factor (PDGF), angiopoietin-1 or conditioned medium, obtained from dermal fibroblast, into the collagen scaffold supported cell ingrowth, migration, differentiation, and the healing process [27,28,29,30,31].

Autologous stem cells, such as bone marrow- or adipose tissue-derived MSCs, are relatively easily available for the colonisation of collagen gels/composites intended as vascular patches, and these cells are able to differentiate into SMCs, as shown in our previous studies and studies by other authors (for a review, see Bacakova et al. 2018) [32]. For stem cell differentiation, specific growth factors and other bioactive molecules can be added in defined doses into the cell culture medium [33,34,35], or these additives can be replaced by a platelet lysate that contains a variety of bioactive compounds, which are physiologically released from platelets during wound healing [36,37]. Moreover, the platelet lysate does not have to be only directly added to the culture medium, but it can also be incorporated into an external controlled drug delivery system, ensuring gradual and long-term release of the bioactive compounds and their prolonged influence on cells.

The novelty of this study therefore lies in preparation of a fully biodegradable nature-derived collagen-based composite, i.e., collagen hydrogel reinforced with collagen particles, incorporated with human adipose tissue-derived stem cells (ASCs) with the aim to differentiate these cells towards SMCs phenotype and to reconstruct the *tunica media* of the physiological vascular wall. For inducing this differentiation pathway, we developed an external drug delivery system, represented by nanofibrous PVA meshes releasing human platelet lysate into the culture medium, or we supplemented the culture medium with a combination of transforming growth factor-β1 (TGF-β1) and bone morphogenetic protein-4 (BMP-4), which has been proven as highly efficient for differentiation of ASCs towards SMCs [34,38]. The cell-material constructs were then evaluated with respect to ASCs proliferation, differentiation, extracellular matrix (ECM) production and remodelling, and mechanical properties of the patches. Finally, the constructs were endothelialised with human umbilical vein ECs in order to reconstruct the *tunica intima*, to mimic a physiological blood vessel wall, and to render the constructs applicable for creating vascular patches and blood vessel replacements. For these purposes, the construct incubated with the platelet lysate-loaded PVA meshes seemed to be the most promising, as indicated by a relatively high degree of ASCs differentiation towards SMCs, a relatively high mechanical stability, low shrinkage of the construct, and its homogeneous and confluent endothelialisation. 

## 2. Results

### 2.1. Properties of Isolated Porcine Collagen

**Figure 1:**
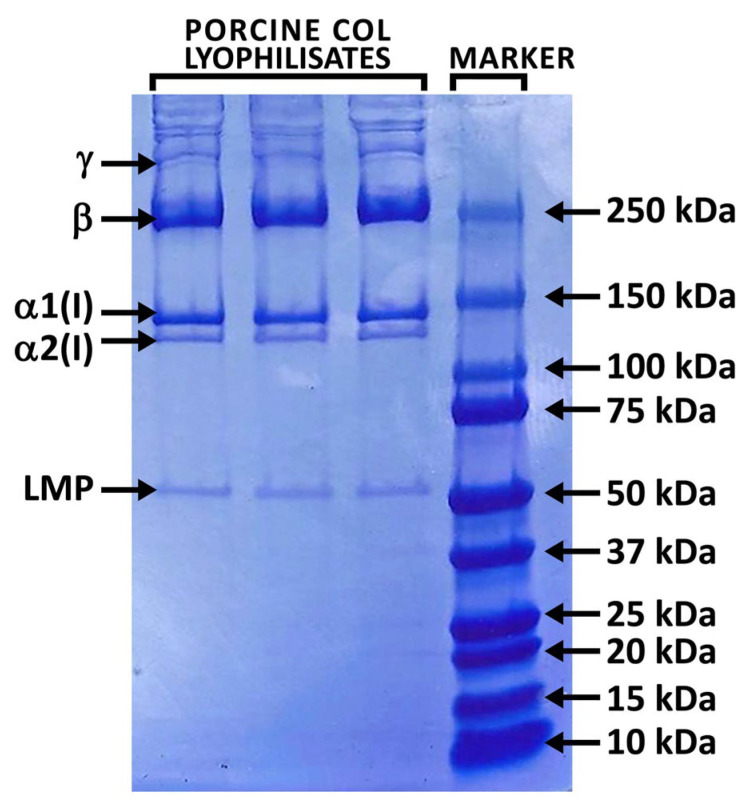
Polypeptide pattern of COL I type isolated from porcine skin.

**Figure 2:**
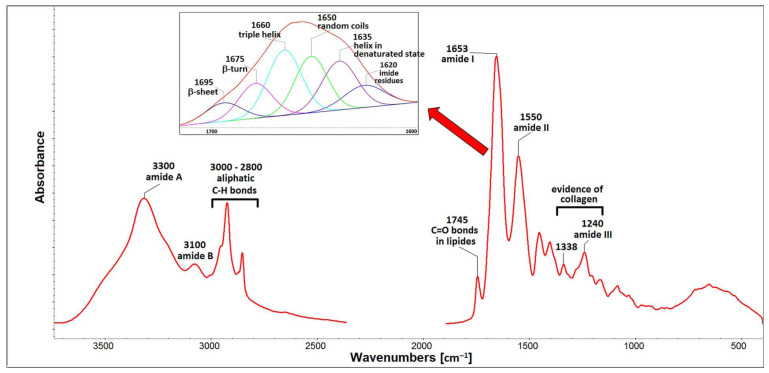
FTIR spectrum of COL I type isolated from pork skin.

Composition of isolated COL is shown in Table 1. COL lyophilizate contains protein, lipids, glycosaminoglycans and water. Total protein content, represented by amino acids (AAs), is 58.5 wt.%. AAs distribution is defined as a number of specific AA residues in 1000 AA units. The Hyp content is reflected in the degree of hydroxylation (40%), which is defined as the ratio of Hyp/(Hyp + Pro). GAGs and lipids were determined close to 4 and 25.5 wt.%, respectively. High scatter of data determined in lipids content can be connected with local inhomogeneity. The amount of interstitial water slightly exceeded 7 wt.%. Figure 1 shows the electrophoretic profiles of porcine collagen. Protein patterns, comprising α1 and α2 chains with an average molecular weight of 130–110 kDa, their dimer (β chain) with molecular weight ∼250 kDa and trimer (γ chain) with molecular weight ∼300 kDa, are apparent. The band intensity ratio of the α1 and α2 chains is approximately 2:1, proving that isolated COL comprise type I existing in two other configurations: α1(I)α2(I)α3(I) and [α1(I)]_2_α2(I) [39]. The similarity in electrophoretic mobility of the α1 chain and α3 chain did not allow for separating them under the electrophoretic conditions [40]. In addition, the profile also comprises a lower molecular weight peptides (LMPs) component visible at 50 kDa. These peptides can be the product of collagen degradation, indicating that, during acidic extraction, some part of collagen (e.g., fragment from the telopeptide regions) may be more susceptible to hydrolysis. LMPs may also include peptide parts of non-collagenous protein residues that may be present in the collagen isolate. The FTIR spectrum of isolated COL can be seen in Figure 2. The spectrum contains five amidic bands, typical for protein, such as amide A at ∼3300 cm^−1^ representing N–H stretching and amide B at ∼3100 cm^−1^ representing stretching vibration of the N–H bonds in secondary amides. The amide I band (∼1650 cm^−1^) originates from C=O stretching vibrations coupled with N–H bending vibration, while the amide II band (∼1550 cm^−1^) arises from N–H bending vibrations coupled with C–N stretching vibrations [41]. Another piece of evidence for the existence of a triple helical structure is the presence of a quartet of bands at ∼1338 cm^−1^ and a triple band at ∼1205, 1240, and 1280 cm^−1^ (amide III) [42]. The amide I region (∼1650 cm^−1^) was deconvoluted into six distinct bands with maxima at ∼1695, 1675, 1660, 1650, 1635, and 1620 cm^−1^. The most dominant components are bands ∼1660 cm^−1^ assigned to the triple helix with contribution from α-helix, and the band at ∼1650 cm^−1^, which corresponds to random coils of α and β chains. Band 1635 cm^−1^ represents a denaturated state. Bands at 1675 cm^−1^ (β-turn), at 1695 cm^−1^ (β-sheet) [43] and band at 1620 cm^−1^ (imide residues) representing other structural states can be associated with the existence of LMP proved by SDS-PAGE. The ATR-FTIR spectrum also contains bands of impurities such as lipids (bands in spectral region 2800–2900 cm^−1^ belong to C-H aliphatic bonds and band at 1745 cm^−1^ assigned to C=O bonds in esters), and low intensity bands in spectral region 900–1100 cm^−1^ relating to GAGs. These residual impurities remain in the collagen after the isolation procedure [42]. Analysis of COL lyophilisates proved that the isolated material is based on collagen with the presence of GAGs, lipids and low molecular weight peptides. These peptides with a lower molecular weight and a shorter chain length than collagen molecules, and with much more hydrophilic and charged amid and carboxyl groups, can stabilize water in a protein gel matrix, as it can be proved by Abdollahi et al. (2018) [44]. 

### 2.2. Morphology of Collagen Fibers and Particles

**Figure 3:**
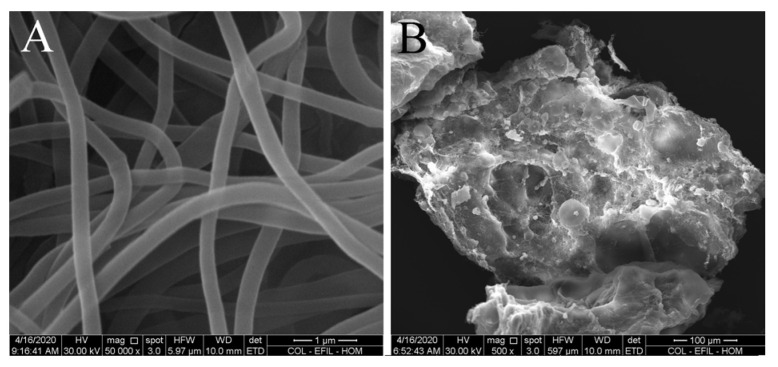
SEM image of electrospun collagen nano/submicrofibers (**A**; mag. 50,000×) and collagen particles prepared via homogenisation of collagen electrospun layers (**B**; mag. 500×).

Figure 3 shows selected images representative of the entire electrospun fibers (mag. 50,000×) and particles prepared via homogenisation of collagen electrospun layers (mag. 500×). Collagen fibers evinced an average diameter of 268 ± 75 nm (*n =* 100). The average length of particles was 363 ± 160 µm (*n =* 100). These measurements confirmed that the collagen fibers were of submicrometer dimensions, but the collagen particles contained both amorphous and fibrous structures. 

### 2.3. Properties of PVA and PVA_PL Meshes 

**Figure 4:**
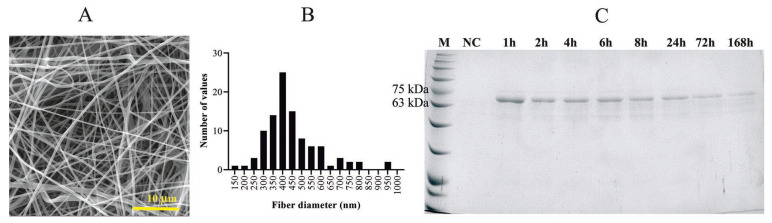
(**A**) SEM image of electrospun PVA with incorporated platelet lysate (PVA_PL), scale bar 10 µm; (**B**) histogram of the fiber diameter distribution in PVA_PL material; (**C**) 10% SDS-PAGE documenting the protein release from PVA_PL after 1, 2, 4, 6, 8, 24, 72 and 168 h in PBS; PVA material without incorporated proteins served as negative control (NC), Coomassie blue staining.

**Figure 5:**
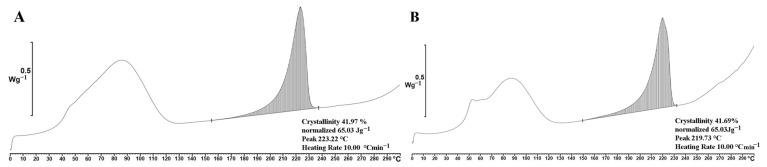
Crystallinity analysis (DSC) of PVA (**A**) and PVA_PL (**B**).

The produced nanofibrous materials from PVA with incorporated proteins (from platelet lysate, PL) have a fiber diameter 455 ± 159 nm with a wide range fiber diameter distribution (Figure 4A,B). The protein content was 19.7 mg per gram of material. The analysis of protein release shows that up to 90% of the protein is released within the first 24 h; the release then slows down and lasts for at least one week (Figure 4C) [45]. Crystallinity analysis did not confirm the influence of incorporated proteins and no significant difference in crystallinity was observed for the materials measured by DSC (Figure 5) and XRD (Figure S1) methods. This means that solubility of PVA was not affected by the incorporation of PL. 

### 2.4. Cell Colonisation of Collagen Gels Reinforced with Collagen Particles 

We developed novel composite scaffolds based on a collagen gel reinforced with embedded collagen particles and compared them with collagen gels without reinforcement. During the first 6 days, the ASCs in collagen without particles were proliferating and reached the highest densities in TGF + BMP and PVA_PL samples (Figures S2 and S3). The TGF + BMP gel evinced increased shrinkage compared to the samples. On day 14, the gels without collagen particles seemed to be degraded and almost disappeared, and further analyses were not performed.

**Figure 6:**
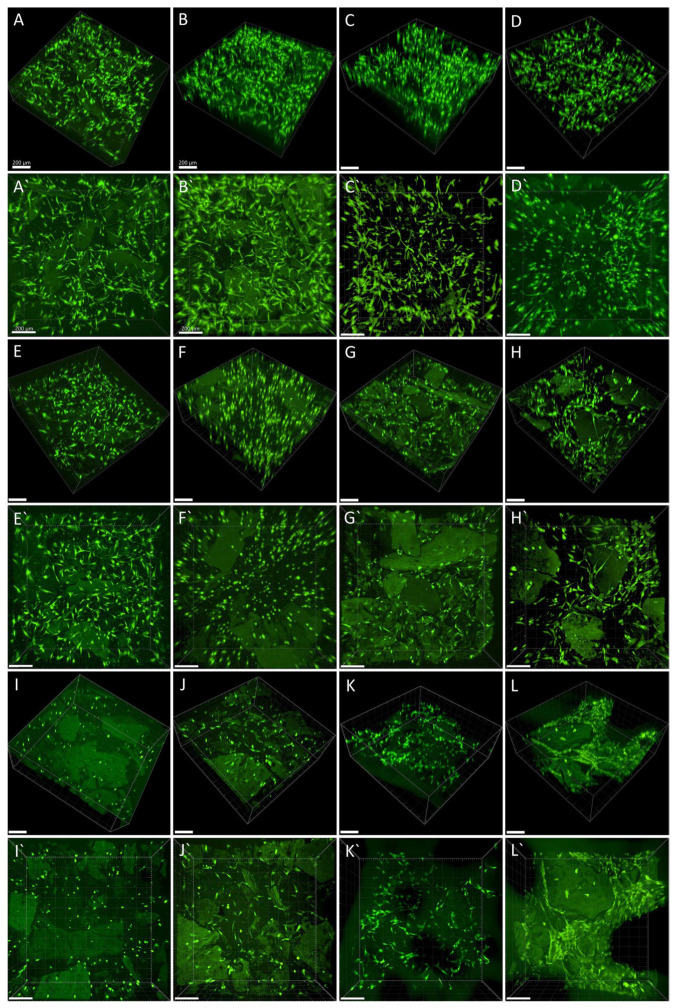
Living ASCs, stained with CellTracker^TM^ Green CMFDA dye, in collagen gel with collagen particles on day 1 (**A**–**D**,**A`**–**D`**), on day 7 (**E**–**H**,**E`**–**H`**) and on day 14 (**I**–**L**,**I`**–**L`**). The cells were cultured in DMEM with 2% of FS (**A**,**A`**,**E**,**E`**,**I**,**I`**), in medium with added PVA nanomats (**B**,**B`**,**F**,**F`**,**J**,**J`**) or with added PVA_PL nanomats (**C**,**C`**,**G**,**G`**,**K**,**K`**) or in medium with TGF-β1 + BPM-4 (**D**,**D`**,**H**,**H`**,**L**,**L`**). Dragonfly 503 (Andor)—spinning disk confocal microscope, obj. × 10, 3D vizualisation (**A**–**L**) and maximum projection (**A`**–**L`**), scale bar = 200 µm, image size 820 × 820 × 500 µm.

**Figure 7:**
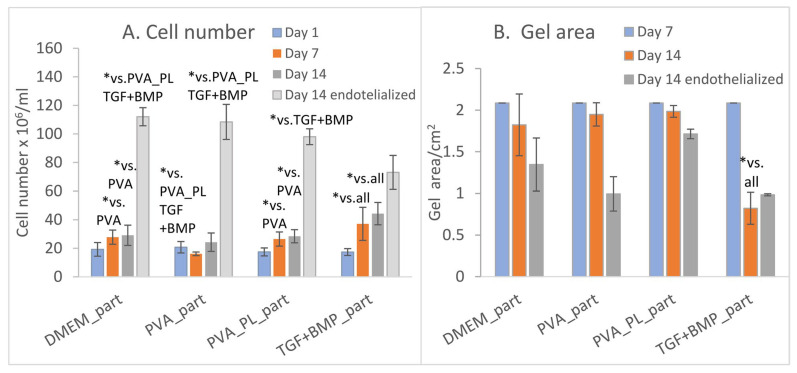
(**A**) Densities of ASCs cultured in collagen gels with particles on days 1, 7, and 14, and density of both ASCs and ECs in endothelialised gels on day 14; (**B**) gel area. Data are expressed as mean ± S.D. Statistical significance is considered * for *p*-value < 0.05.

In the experiments with collagen gels with collagen particles, we observed proliferation and differentiation of ASCs incorporated into these scaffolds and cultivated in the presence of platelet lysate-loaded nanomats (PVA_PL_part samples). Cultivation of cells in gels in standard DMEM media and in media with PVA mats without PL served as negative controls (DMEM_part and PVA_part samples). Conversely, the addition of TGF-β1 and BMP-4 to the media represented a positive control for the cell differentiation towards SMCs (TGF + BMP_part). The process of gel preparation with ASCs previously stained with CellTracker^TM^ Green CMFDA dye was relatively fast, and thus this prevented cells sedimentation and allowed for initial homogeneous cell distribution in the collagen gel. Collagen particles had a weak autofluorescence, and therefore they were partially visible on confocal images. The prepared samples showed a similar morphology and population density of ASCs on day 1 (Figure 6A–D,A`–D`). On day 7, the population density of ASCs was highest in TGF + BMP_part sample and lowest in the PVA_part sample (Figure 6A,E–H,E`–H`). The cells grew both in the gel and on the surface of the particles. The ASCs further increased their population densities on day 14; the highest value was observed in the TGF + BMP_part sample (Figure 6I–L,I`–L` and Figure 7A). Seeding ECs on the top of the ASC-incorporated gels enhanced the proliferation of both ASCs and ECs. Surprisingly, the highest cell population density on day 14 was observed in DMEM_part sample, which was used as a negative control (Figure 7A). 

Collagen particles hindered the measurement of the gel depth via microscopic evaluation. Thus, in order to evaluate the gel shrinkage, the gel area was measured inside cell culture dishes. There was no shrinkage of gels on day 7 (Figure 7B). On day 14, the shrinkage of ASCs-seeded gel was apparent only in the TGF + BMP_part sample. All the samples seeded with both ASCs and ECs shrank until day 14; a slightly higher extent of shrinkage was in the TGF + BMP gel. The shrinkage was probably caused by a higher proliferation rate of both ASCs and ECs in EGM-2 medium from day 8. The presence of PVA_PL system seemed to prevent the gel shrinkage, although the cell density was quite high. 

**Figure 8:**
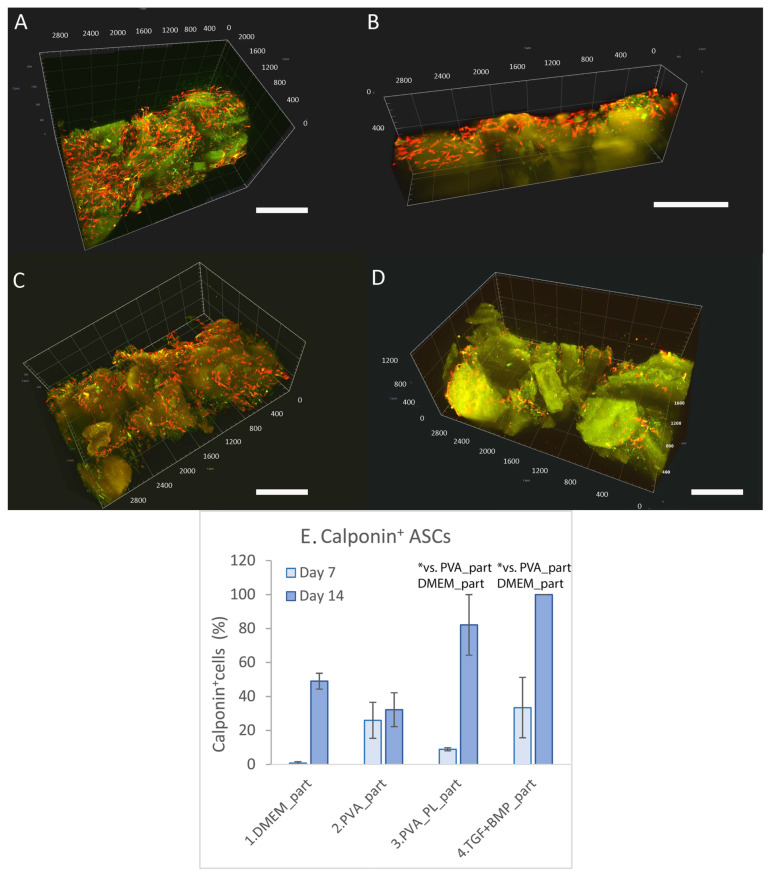
Immunofluorescence staining of alpha-actin (red) and calponin (green) in ASCs in collagen gels with collagen particles on day 7. The cells were cultured in DMEM with 2% of FS (**A**), in medium with added PVA nanomats (**B**), with added PVA_PL nanomats (**C**) or in medium supplemented with TGF-β1 + BPM-4 (**D**), scale bar = 800 µm. Percentage of calponin^+^ cells in collagen gels on days 7 and 14 (**E**). Cell nuclei were counterstained with DAPI. Zeiss Z.1 lightsheet microscope, obj. × 10 for excitation, obj. × 20 for detection, zoom × 0.4 tile scans. Data are expressed as mean ± S.D. Statistical significance is considered for * *p*-value < 0.05 in comparison with the sample of the same number, and is depicted above the graph columns.

ASCs differentiation towards SMCs, which are an essential cellular component of vascular wall, was evaluated by immunofluorescence staining of alpha-actin and calponin, an early marker and a mid-term marker of differentiation into smooth muscle cells. On day 6, the DMEM gel without particles contained cells positive for alpha-actin, but the cells were negative for calponin (Figure S4). The PVA samples contained both alpha-actin positive cells and a small portion of calponin-positive cells. On the other hand, both PVA_PL and TGF + BMP samples contained alpha-actin-positive cells and a high portion of calponin-positive cells. On day 7, staining of reinforced gels showed the ASCs positive for alpha-actin, but only a small portion of the cells were positive for calponin (Figure 8). On day 14, the highest ratio of calponin-positive cells was in both PVA_PL_part and TGF + BMP_part gels on day 14 (Figure 8C). In the latter sample, all ASCs were positively stained for calponin. 

**Figure 9:**
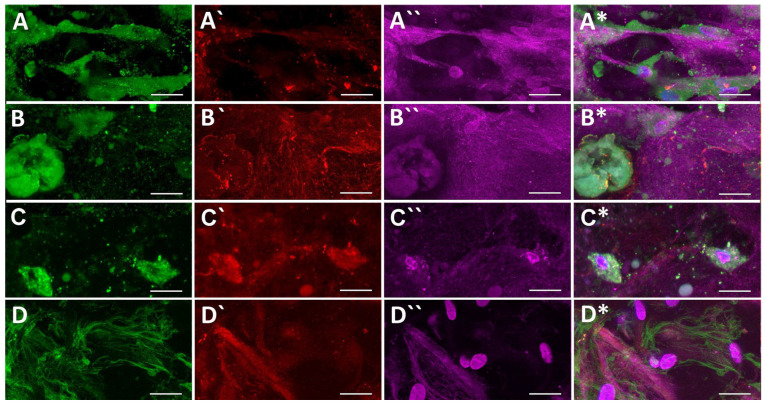
Immunofluorescence staining of fibronectin (green, **A**–**D**,A*–D*), type I collagen (red, **A`**–**D`**,A*–D*), second harmonic generation signal of fibrous collagen (purple, **A``**–**D``**,A*–D*) in ASCs in collagen gel. The cells were cultured in DMEM with 2% of FS (**A**,**A`**,**A``**,A*), in medium with added PVA nanomats (**B**,**B`**,**B``**,B*), with added PVA_PL nanomats (**C**,**C`**,**C``**,C*) or in medium with TGF-β1 + BPM-4 (**D**,**D`**,**D``**,D*) for 7 days. Zeiss, LSM 780 microscope system, obj. × 63×, image size 135 µm × 67 µm, scale bar = 25 µm.

The collagen gels with particles seeded with ASCs were also evaluated for ECM proteins fibronectin and type I collagen by immunofluorescence staining and SHG microscopy (Figure 9). Fibronectin (green) was observed as filamentous only in the TGF + BMP_part samples. In the other samples, the signal was present mainly inside cells or in their vicinity, and was diffuse or granular. Type I collagen was detected by immunofluorescence staining (red), which visualizes total collagen, and also by the SHG technique (purple), which visualizes mature collagen organised into fibrils. In PVA_PL_part samples, the immunofluorescence of collagen was localised predominantly around the cells, which suggest de novo formation of collagen by these cells. SHG staining was also brightest in PVA_PL_part samples, but fibrous arrangement of collagen was most prominent in TGF + BMP_part samples. At the same time, however, the TGF + BMP_part samples contained a relatively low amount of collagen, especially around the cells, which suggested collagen remodelling, particularly its reorganisation and breakdown. However, the quantitative analyses of immunofluorescence signal of both fibronectin and type I collagen did not reveal any differences among the tested samples. 

**Figure 10:**
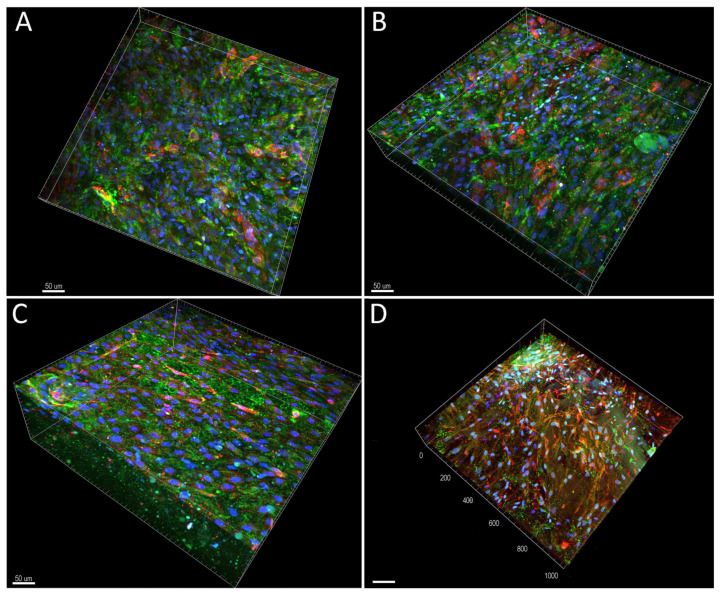
Immunofluorescence staining of von Willebrand factor in ECs (green) and alpha-actin (red) in ASCs in collagen gel reinforced with collagen particles cultured in DMEM with 2% of FS (**A**), with added PVA nanomats (**B**), with PVA_PL nanomats (**C**) or in medium with TGF-β1 + BMP-4 (**D**) for 8 days and, subsequently, in EGM-2 medium, for 6 days. The cell nuclei are counterstained with Hoechst, Bruker Ultima, obj. × 25, scale bar = 50 µm.

Endothelialisation of the collagen gels reinforced with particles and incorporated with ASCs was performed with ECs from day 8 to day 14 of the culture. The DMEM_part, PVA_part, and PVA_PL_part samples were homogeneously endothelialised with almost confluent EC layer, as it was proved by staining of the von Willebrand factor (Figure 10A–C). In contrast, the TGF + BMP_part sample was not covered with ECs homogeneously, i.e., these cells tended to form separate dense islands, and the staining of von Willebrand factor seemed to be weaker (Figure 10D). However, the quantification of fluorescence signal of area occupied with von Willebrand factor and the intensity of staining of von Willebrand factor normalised to cell nuclei did not show any significant differences among the tested samples. In all samples, the ECs were localised on the gel surface, but sometimes they were found inside the collagen gel as well. ASCs were found freely distributed in collagen gel or adhering on collagen particles. The ability of collagen particles to be colonised with ASCs illuminates a slower collagen degradation and sample shrinkage, which were enormous in gels without reinforcement. The submicro-/nano-structure and crosslinking of the particles might contribute to their anti-shrinkage effect inside the collagen gel, which further increased with the presence of PVA_PL nanomeshes in cell culture medium. 

### 2.5. Proteomics of the Cell-Material Constructs

A total of five groups, namely collagen gels with ASCs immediately after preparation (D0), and collagen samples after 12 days (D12) in culture, i.e., DMEM_part, PVA_part, PVA_PL_part, and TGF + BMP_part containing ASCs and ECs, were analysed by mass spectrometry according to their protein concentration. There was a high number of significant changes detected between the control group (D0) and the samples after a 12-day cultivation, i.e., DMEM_part/D0 = 731 significantly upregulated proteins (s.u.p.) for a 12-day cultivation group; PVA_part/D0 = 718 s.u.p.; PVA_PL_part/D0 = 756 s.u.p.; TGF + BMP_part/D0 = 895 s.u.p.). 

Because the number of significantly altered proteins was too high, we selected three protein groups, i.e., proteins involved in cell differentiation (Table 2), ECM (Table 3) and proteins associated with ECM remodelling (Table 4) for better comprehensibility. 

#### 2.5.1. Proteins Involved in Cell Differentiation

Table 2 displays 25 significantly upregulated proteins between day 12 and day 0 (D12/D0 cultivations) involved in cell differentiation. They comprise proteins related to SMCs differentiation (i.e., calponin and caldesmon, alpha-parvin, myosin phosphatase Rho-interacting protein), to muscles and vessels (cofilin-2, tropomyosines, plectin, transgelin, tropomodulin-3, nexilin, utrophin, filamin-A, filamin-C, alpha-adducin, fascin, myosin light polypeptide 6, myosin phosphatase Rho-interacting protein), proteins related to endothelial cells (i.e., platelet endothelial cell adhesion molecule, von Willebrand factor, endothelial monocyte-activating polypeptide 2, endothelial differentiation-related factor 1), proteins present in epithelial cell types (i.e., LIM and SH3 domain protein 1, alpha-actinin-4, F-actin-capping protein subunit alpha-1) and proteins that influence the differentiation of various other cell types (vinculin, fascin).

The increased concentration of caldesmon was in both PVA_PL_part and TGF + BMP_part samples compared to PVA_part sample. The TGF + BMP_part samples contained significantly increased concentrations of myosin phosphatase Rho-interacting protein, transgelin, and tropomyosin alpha-1 chain. On the contrary, higher concentrations of both nexilin and fascin were found in the PVA_part compared to TGF + BMP_part samples. 

#### 2.5.2. ECM Proteins

There were detected 19 significantly upregulated proteins between day 12 and control D0 sample in the ECM group (Table 3). The ECM group involves laminins (subunit alpha-2, alpha-4, beta-1, and gamma-1); collagens (type VII and triple helix repeat-containing protein 1, collagen alpha-1(V) chain, and collagen alpha-1(XII) chain), fibronectin, basement membrane-specific heparan sulfate proteoglycan core protein, decorin, tenascin, thrombospondin-4, lumican, fibulin-1, fibrilin, extracellular matrix protein 1, nidogen, and galectin-3-binding protein. Among the samples, the cells in TGF + BMP_part sample produced the significantly highest amounts of both type VII collagen and tenascin. 

In both PVA-part and PVA_PL_part, the cells expressed more extracellular matrix protein 1, galectin-3-binding protein, laminin subunit alpha-4, nidogen-1 and thrombospondin-4 compared to the TGF + BMP_part. 

#### 2.5.3. Remodelling Proteins

Among ECM remodelling proteins, there were detected 13 significantly upregulated proteins (D12/D0 cultivations) in DMEM_part, PVA_part, PVA_PL_part and TGF + BMP_part between day 12 and control D0 group (Table 4). This remodelling group consists of five matrix metaloproteinases (MMP1, MMP2, MMP14, disintegrin and metalloproteinase with thrombospondin motifs 1), three enzymes required for collagen crosslinking or its regulation (lysyl oxidase homolog 2, procollagen-lysine 2-oxoglutarate 5-dioxygenase 1 and 2, and periostin), two transforming growth factor beta-related proteins (TGF-1-induced transcript 1 protein and TGF-beta-induced protein ig-h3) and metaloproteinase inhibitors (TIMP1 and procollagen C-endopeptidase enhancer 1). In TGF + BMP_part, higher amounts of periostin, matrix-remodelling-associated protein 7 (MXRA7) and transforming growth factor beta-1-induced transcript 1 protein (TGFB1I1) were measured compared to all other groups (for periostin), compared to PVA_part (for MXRA7) or compared to PVA_PL_part (for TGFB1I1). The expression of interstitial collagenase MMP1 was higher in both PVA_part and PVA_PL_part samples in comparison with the TGF + BMP_part.

### 2.6. Biomechanical Properties

**Figure 11:**
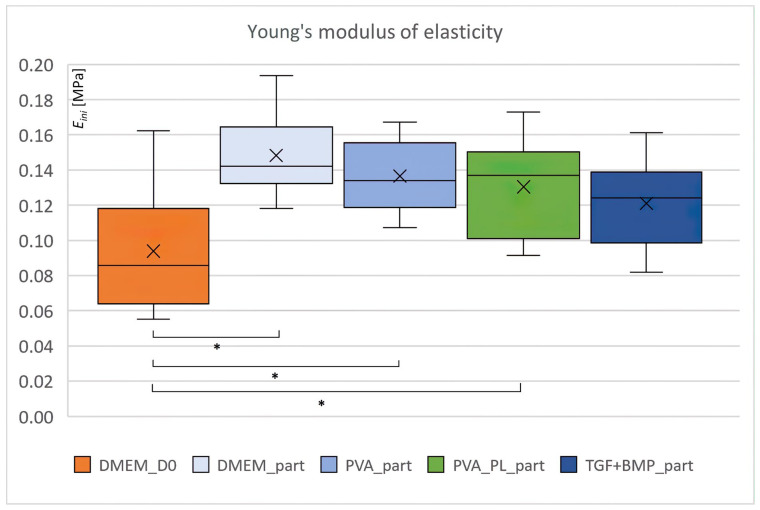
Initial Young modulus of elasticity of reinforced collagen gel with ASCs immediately after preparation, i.e., DMEM_D0, and on day 5 after seeding, i.e., DMEM_part, PVA_part, PVA_PL_part and TGF + BMP_part. box plot diagram, * denotes statistically significant differences (Student’s *t*-test, 0.05).

The mechanical properties of the composite material were characterised by the initial Young modulus of elasticity, total deformation energy and the ratio of elastic energy, i.e., the ratio between the elastic deformation energy and the total deformation energy. Young modulus (*E_ini_*) was higher in all collagen samples after 5 days in culture compared to the DMEM sample immediately after preparation (signed DMEM_D0; see Figure 11). The statistically significant differences (Student’s *t*-test, 0.05) were observed for DMEM, PVA and PVA_PL samples. The stiffness of the samples increased by more than 39% after 5 days of cultivation. This result suggests remodelling of the gel by ASCs. Other biomechanical parameters, i.e., total deformation energy (*U_c_*) and ratio of elastic energy (*U_el_/U_c_*), were similar for all samples (see Figures S5 and S6 in the Supplementary File). No statistically significant differences were observed.

## 3. Discussion

Cardiovascular patches and prostheses based on collagen can be prepared using various methods, such as collagen gel casting, gel compression, 3D printing, decellularisation of tissues, etc. [46]. These implants can be used as acellular or can be cellularised either by conventional in vitro seeding cells on the prepared materials or by direct admixing the cells into the matrices during their preparation. Both of these approaches were used in the present study, i.e., the collagen gels were directly incorporated with ASCs and after the pre-differentiation of these cells towards SMCs, the constructs were seeded with ECs.

### 3.1. Collagen Gel Preparation, Gel Reinforcement 

Collagen gels with entrapped cells can be easily prepared using a wide range of collagen concentrations. The main disadvantage of a collagen gel incorporated with cells is contraction of this gel, which is more intense in gels with a lower initial collagen concentration. Collagen contraction/shrinkage stimulated the cell apoptosis and reduced the synthesis of ECM proteins [47]. In our experiment with collagen reinforced with collagen submicrometer particles, we used a final collagen concentration of 3.79 mg/mL, but this collagen contained about 25% of GAGs and lipids. We observed only a limited gel contraction after its incorporation with ASCs, and this contraction was observed predominantly in gels incubated in medium with TGF-β1 + BMP-4 and in endothelialised gels. Another disadvantage of a collagen gel is weak mechanical resistance of the gel. To overcome it, the gel can be reinforced with fibers, particles or by preparation of various composites with other materials. For example, Nashchekina and colleagues [48] prepared polylactide scaffolds filled with a collagen gel of collagen density in the range from 1 to 3.5 mg/mL, colonised with mesenchymal stem cells (MSCs). The collagen density influenced the cell morphology, which was more spindle-shaped in gels with a density of 2 mg/mL or higher. This composite stimulated the production of laminin and fibronectin, especially from day 10 [48]. Similarly, fibronectin, laminin, and a number of other ECM proteins were produced by a co-culture of ASCs and ECs in our collagen gels until day 12. Collagen gel reinforced with fibrin-coated nanofibrous membrane and cellularised with fibroblasts immigrating from the membrane into the gel was developed as a skin construct [49]. This system was also advantageous in preventing the undesired gel shrinkage because the cells gradually colonizing the gel from the underlying membrane caused a significantly lower contraction of the gel than the cells admixed directly into the material [49]. Collagen gel was also reinforced by polymerisation with polyethylene glycol (MW  =  8000) [50] or by crosslinking with transglutaminase [51]. 

### 3.2. Cellular Component of the Gel, Cell Differentiation 

For cardiovascular tissue engineering, the collagen gel should contain suitable cell components. ASCs are able to differentiate into smooth muscle phenotypes in cell culture media supplemented with various growth factors, such as TFG-β1 with ascorbic acid [35,52], using various combinations of vascular endothelial growth factor (VEGF), fibroblast growth factor (FGF), platelet-derived growth factor (PDGF), TGF-β and B27 minus insulin supplement for inducing either synthetic or contractile phenotypes of SMCs differentiating from human induced-pluripotent stem cells [33]. Other factors inducing SMC differentiation include TGF-β3, sphingosylphosphorylcholine [53], and BMP-4, especially in combination with TGF-β1 ([34]. In a study by Elçin and colleagues [38], the combination of TGF-β1 with BMP-4 was selected as the most efficient among the media supplemented with TGF-β1 alone, with BMP-4 alone, with angiotensin II (Ang II), Ang II + TGF-β1 and with Ang II + BMP-4, and thus this medium was also used in our present study as a positive control for ASC differentiation towards SMCs. The biochemical signal inducing the SMC differentiation, provided by various culture media supplements, can be further enhanced by a mechanical stimulation. For example, a combination of PDGF-AB and TGF-β1 with cyclic stretching considerably enhanced the differentiation of ASCs towards SMCs [35]. 

In our study, the ASCs were cultured in DMEM medium with 2% of FS and ascorbic acid, further supplemented either with direct addition of TGF-β1 + BMP-4 or of an external drug delivery system, based on PVA nanofibers releasing platelet lysate (PVA_PL). Moreover, for co-culture of ASCs inside the material with ECs on its surface, the cell culture medium contained growth factors, such as VEGF, FGF-2, insulin-like growth factor-1 (IGF-1), epidermal growth factor (EGF) and heparin, from which VEGF with heparin were observed to stimulate the differentiation of ASCs towards SMCs in our earlier study [28]. In our collagen gels, we proved the presence of alpha-actin and calponin, i.e., markers of SMC differentiation, in ASCs cultured with TGF-β1 + BMP-4 supplement and with the external PVA_PL drug delivery system.

In addition, ASCs have the capacity to mediate wound healing through mitochondrial transfer and the paracrine secretion of various growth, antiapoptotic, angiogenic, antioxidant, anti-inflammatory and immunomodulatory factors, contained in exosomes and extracellular vesicles (for a review, see [32]). Furthermore, exosomes from ASCs can be internalised by other cell types, e.g., fibroblasts to stimulate the cell migration, proliferation and collagen synthesis in a dose-dependent manner, and to significantly accelerate cutaneous wound healing [54]). ASCs co-cultured with monocytes enhanced ECM deposition in comparison with the ASCs in monoculture, which had been differentiated towards SMCs using supplementation with both TGF-β1 and retinoic acid. We co-cultured ASCs in the collagen gel with endothelial cells that are naturally present in blood vessels. In our co-culture experiment, the TGF + BMP_part sample supported the differentiation of ASCs towards SMCs well but did not support homogeneous endothelialisation of the gels with ASCs. It could be explained by a dual effect of TGF-β1 and BMP-4, which can either stimulate or inhibit the migration and proliferation of ECs. TGF-β1 has multiple functions: it controls embryonic development, cell proliferation, migration and differentiation, ECM production and new blood vessels formation. TGF-β1 can stimulate the proliferation of various cell types, such as mesenchymal stem cells, fibroblasts, chondrocytes, osteoblasts and endothelial cells, but it can also inhibit the cell proliferation, including the proliferation of endothelial cells, epithelial cells and keratinocytes. This is due to the fact that TGF-β1 can activate two distinct type I receptor/Smad signalling pathways with opposite effects: the TGF-beta/ALK1 pathway induces endothelial cell migration and proliferation, necessary for the blood vessel formation, while TGF-beta/ALK5 pathway leads to inhibition of cell migration and proliferation and contributes to the blood vessel maturation [55,56]. TGF-β1 together with VEGF is also involved in the angiogenic response to hypoxia, and their response is connected with NOX4-mediated reactive oxygen species production [57]. BMP-4 also has a dual (controversial) effect on the migration and proliferation of ECs. In a study by [58], the addition of BMP-4 into the culture medium significantly increased migration and proliferation of mouse embryonic stem cell-derived ECs and human microvascular ECs via activation of VEGF and angiopoetin signalling pathways. However, in a recent study by [59], the addition of BMP-4 into cell culture medium inhibited the migration of human umbilical vein ECs, which was attributed to the accumulation of reactive oxygen species (ROS), induced by BMP-4. 

In samples exposed to the external drug delivery system consisting of PVA meshes slowly releasing platelet lysate, we achieved not only the differentiation of ASCs towards SMCs, but also a homogeneous and almost confluent endothelialisation. Platelet α-granules contain a wide variety of bioactive molecules that are released upon activation via physical or physiological stimuli and participate during wound healing, such as coagulation factors (e.g., factor V), adhesion molecules (e.g., von Willebrand factor, fibrinogen, thrombospondin), platelet factor 4 (PF-4), protease inhibitors (α2-macroglobulin, α2-antiplasmin), plasma proteins (IgG, albumin), proteoglycans, EGF, heparin-binding EGF-like growth factor, IGF-1, transforming growth factors (TGF-α, TGF-β), platelet-derived growth factors (PDGF-AA, PDGF-AB, PDGF-BB), soluble CD40L, immunoglobulins such as vascular cell adhesion molecule-1 (VCAM-1), intercellular adhesion molecule-1 (ICAM-1), chemokine (C-C) ligand 5 and chemokine (C-X-C) ligand 1/2/3 [36]. The growth factors released from platelets act in the inflammatory phase of wound healing by stimulating the chemotaxis of monocytes to the site of injury and their differentiation into macrophages [37]. EGF has been observed to stimulate proliferation of fibroblasts, keratinocytes, and vascular ECs, and the production of fibronectin [60]. PDGF-BB is a potent stimulant for vascular SMC proliferation, migration, phenotypic modulation, and for pericyte recruitment; it prevents aberrant angiogenesis, reduces circumferential enlargement of vessels and supports vascular splitting into functional capillary network even with high VEGF concentrations [61,62]. In our earlier study, the PVA_PL nanofibers were able to release PL for more than for 7 days in sufficient concentration to stimulate growth of ECs, HaCaT keratinocytes and dermal fibroblasts [63]. Similarly, in this study, the slow and long-term release of PL from PVA meshes had a clearly positive effect on cell performance, manifested by the differentiation of ASCs towards SMCs, and particularly by the formation of a continuous EC layer. 

Similarly, in studies by other authors, ASCs cultured in a medium supplemented with 5% of platelet lysate and without FS created more homogeneous phenotype, had a high proliferative capacity, secreted various proteins, such as basic fibroblast growth factor (bFGF), interferon-γ (IFN-γ), and IGF-1, and had immunomodulatory effects [64]. ASCs cultured in LaCell StromaQual^TM^ medium supplemented with 1% human platelet lysate enhanced proliferative capacity by about 70% compared to supplementation with 10% of FS. The ability to form colonies (evaluated by a colony-forming unit-fibroblast assay, CFU-F) was, however, reduced with human PL [65]. In our experiments, the external PVA_PL delivery system stimulated preferential production of ECM matrix proteins laminin, thrombospondin-4, galectin-3-binding protein, extracellular matrix protein 1 and interstitial collagenase (MMP1), while the TGF-β1 + BMP-4 supplement supported synthesis of matrix-remodelling-associated protein 7, periostin, TGF-β1-induced transcript 1 protein, tenascin, and type V and VII collagens. On the other hand, many other proteins, e.g., fibronectin, decorin, vinculin, and lumican, were produced equally in both types of samples. 

### 3.3. Mechanical Properties of the Constructs

Mechanical properties of cardiovascular patches are important for further clinical use. Biomechanical evaluation of our samples was performed on day 5 of static culture, as the construct had a proper volume for testing via an unconfined compression test. Despite this short time interval, there was an increase in the initial Young modulus of elasticity in all samples compared to the D0 time interval, although there were no differences among the groups. Our gels contained 0.5 × 10^6^ ASCs cells/mL of the gel. Camasão and colleagues [66] have observed that higher initial SMCs densities during collagen gel preparation, i.e., 1.5–4.5 × 10^6^ cells/mL, increased the initial collagen gel compaction/shrinkage, but did not negatively influence the long-term collagen gel compaction. In the samples with high cell densities, the cells produced a higher amount of collagen than the samples seeded with a lower cell density (0.5 × 10^6^ cells/mL). The initial (E_0_) and equilibrium elastic modulus (E_E)_ for the construct with 4.5 × 10^6^ cells/mL were 220 ± 18 kPa and 58 ± 4 kPa, respectively, and these values were about two- and threefold higher compared to the gel with the lowest cell seeding density. At the same time, on day 7, the E_E_/E_0_ ratio, which expresses an elastic component, was greater in the high cell seeding density sample [66]. For comparison, the initial Young modulus of elasticity of our samples was in the range from 121 ± 24 kPa to 148 ± 24 kPa for TGF + BMP_part and DMEM_part samples after 5 days of culture, and the increase was in the range from 29 to 57%, respectively. The ratio of elastic energy was slightly higher for the PVA_PL_part sample. In the PVA_PL_part sample, upregulated protein interstitial collagenase (MMP1) was detected, thus collagens were probably more cleaved and loosen up there. The PVA_PL_part sample also evinced upregulated protein thrombospondin, which is a modulator of elastic fiber organisation [67]. TGF + BMP_part had a slightly higher total deformation energy than the PVA_PL_part, which was probably due to a significantly higher concentration of several types of collagens (V, VII, XII) detected by proteomic analysis. In addition, significantly upregulated protein periostin in the TGF + BMP_part sample could play a positive role in the contractility of the ECM scaffold. Periostin knockout −/− fibroblasts showed a significantly reduced ability to contract a collagen gel [68]. TGF + BMP_part also contained a significantly higher concentration of protein tropomyosin alpha-1 chain, which is involved in the actin contractile system, and protein transgelin, which is a shape-change sensitive actin crosslinking protein [69]. The elastic modulus of collagen hydrogel reinforced with silk fibroin, measured by a compression method, increased from 1.6 kPa for pure collagen to 2.6 kPa or 2.3 kPa for 50% and 100% silk content, respectively [70]. The gels containing 50% and 100% of silk fibroin reinforcement allowed for the growth of bone marrow MSCs. Improving the mechanical properties of collagen gel allows for its further processing by 3D printing for cardiovascular patches development. In another study [71], the addition of PVA/liposome nanofibers into the hyaluronate/type I collagen/fibrin composite gel increased gel stiffness and Young modulus. In addition, these nanofibers served as a carrier of bFGF and insulin for osteochondral regeneration. In another study, a texture reinforcement prepared from collagen fibers, which had been isolated from a coral, improved mechanical properties of crosslinked alginate gel dramatically [21]. The tubular construct evinced radial compliance values as follows: 9.97 ± 3.80, 4.88 ± 0.99, and 3.38 ± 0.64%/100 mmHg for the pressure ranges of 50–90, 80–120, and 110–150 mmHg. These values are similar to those of young and old coronary arteries. Another ureteral tubular collagenous scaffold was reinforced with Vicryl mesh [72], loaded with heparin, FGF-2 and VEGF, and then implanted successfully into pigs. In a study of Syedain and Tranquillo [73], TGF-β1 stimulated collagen production by neonatal human dermal fibriblasts during the first two weeks of cyclic stretching of fibrin-based tubular structures. At 5 and 7 weeks, however, both ultimate tensile strength and collagen concentration were lower, and elastin concentration was higher in the samples cultured with TGF-β1 [73]. Static culture of pure collagen gel with embedded SMC without any reinforcement did not improve mechanical properties of collagen gel. However, the samples evinced the improved initial modulus and relaxed modulus after 5 days of 10% cyclic strain loading at 0.5 Hz. In addition, mechanical loading caused gel contraction [74].

## 4. Materials and Methods

### 4.1. Isolation of Collagen

The type I collagen (COL), used for preparing the hydrogel matrix, was isolated from porcine skin (Czech Improved White pig, 6 months old, the skin was obtained from the slaughterhouse) using the following protocol: incubation in 70% ethanol (*v/v*) solution (1 g skin/10 mL, 30 min), washed 3× by water and subsequently exposed to an acetic acid solution in ratio 1:1000 (*v/v*), 1 g of COL/20 mL for 48 hrs). COL in collected supernatant was precipitated using 0.1 M NaOH solution in ratio 6:1 (*v/v*) up to slightly neutral pH 6–7. The obtained pellets were then dissolved again in acetic acid solution 1:1000 (*v/v*), frozen to −30 °C and lyophilised. All isolates were stored in a freezer at −20 °C.

### 4.2. Preparation of Collagen Submicrometer Fibers and Particles

Collagen fibers were used as a reinforcement for optimisation of mechanical properties of the collagen hydrogels. Fibers based on collagen (type I, VUP Medical, Brno, Czech Republic) were prepared via the electrospinning (4SPIN, Contipro, Dolní Dobrouč, Czech Republic) of an 8 wt% collagen PBS/ethanol solution modified by 8 wt% (to collagen) polyethylene oxide (PEO; Mr. 900,000, Sigma-Aldrich, St. Louis, MO, USA). The stability of all the collagen layers was enhanced by means of crosslinking with a 95% ethanol solution containing *N*-ethyl-*N*′-(3-(dimethylaminopropyl)carbodiimide (EDC) and *N*-hydroxysuccinimide (NHS) at a weight ratio of 4:1 (both Sigma-Aldrich, St. Louis, Missouri, USA), for 24 h at 37 °C. Cross-linked layers were further washed in 0.1 M Na_2_HPO_4_ (2 × 45 min), and by rinsed using deionised water (30 min). In this step, PEO was fully leached out [75]. The materials were then frozen at −30 °C for 5 h and lyophilised. Collagen fibers were then swelled in ethanol (100%), and their homogenisation, resulting in particle formation, was achieved using a disintegrator (10,000 rpm, 10 min), followed by rinsing with deionised water (30 min), freezing at −30 °C and lyophilising.

### 4.3. Analyses of Porcine Collagen Composition

Composition of isolated COL lyophilisates was studied by various analytical methods. The determination of interstitial water (directly bound to the triple-helix) was performed according to the standard ISO 6496:1983 (Animal feeding stuffs—Determination of moisture content), i.e., by drying to 160 ± 2 °C for 4 h. Amino acid analysis was performed using an Ingos AAA 400 analyser (INGOS s.r.o., Prague, Czech Republic). The hydroxyproline (Hyp) content was determined according to the ISO 3496:1994(E) standard (Meat and Meat products—the determination of hydroxyproline content). Content of lipids was assessed by the Schmidt-Bondzyński–Ratzlaff method, according to Czech technical standard EN ISO 1735:2004. Total content of glycosaminoglycans (GAGs), i.e., long unbranched polysaccharides, was quantified by high-performance liquid chromatography (HPLC) method based on hexosamines; i.e., glucosamine and galaktosamine, due to the formation of fluorescent N-acetylated hexosamine derivatives by reaction with a specific derivatisation agents—*o*-phthaldialdehyde [76]. Sodium dodecyl sulfate–polyacrylamide gel electrophoresis (SDS-PAGE) was performed using the Mini-Protean Tetra Cell electrophoretic system from BIO-RAD on a gradient TGX Miniprotean Precast Gel, 4–15% (BIO-RAD, Hercules, CA, USA). The secondary structure of the isolated COL was evaluated by means of infrared spectrometry (FTIR) using iS50 infrared spectrometer (Nicolet Instrument, Madison, WI, USA) by attenuated total reflection (ATR) mode (GladiATR, PIKE Technologies, Madison, WI, USA) with a diamond crystal. Obtained spectra were processed by OMNIC version 9 software (Thermo Fisher Scientific, Waltham, MA, USA). Detailed information relating the used analytical methods can be found in a study by Stepanovska et al. [77].

### 4.4. Morphology of Collagen Submicrometer Fibers and Particles

The morphology of electrospun submicrometer-scale fibers and particles were characterised using scanning electron microscopy (SEM) (QUANTA 450, FEI). Randomly selected images representative of the entire electrospun fibers (mag. 50,000×) and homogenised particles (mag. 100×) were used to measure the diameters of 100 individual fibers and/or particles using the ImageJ software (US National Institutes of Health, Bethesda, MD, USA, http://imagej.nih.gov/ij/ (accessed on 30 August 2022).

### 4.5. Platelet Lysate Preparation

Platelet lysate (PL) was prepared from platelet-rich solution, which was obtained from healthy blood donors (from Regional Hospital Liberec, Czech Republic), and all blood donors signed the informed consent. Concentration of platelets was 843 × 10^6^ per mL. Fresh platelet-rich solution was frozen at −80 °C at least for 24 h, and then thawed over night at 4 °C (causing gentle cell lysis). Then, the cell lysate was centrifuged (1000× *g*, 30 min), and the supernatant after centrifugation (PL) was stored at 4 °C until use (not longer than 24 h) [45].

### 4.6. PVA_PL Nanofibrous Mat Preparation

Nanofibrous mats were prepared from 10% solution of PVA (molecular weight 125,000, degree of hydrolysis 98–98.9%, Mowiol 20–98, Merck Spol. S.R.O., Prague, Czech Republic) in water: ethanol (9:1) solvent system. In case of protein loaded mats, the 10 g of platelet lysate was added into 90 g pre-PVA solution 30 min before electrospinning and gently mixed at room temperature until use (for details, see [45]). Materials were then electrospun using Nanospider™ 1WS500U machine (Elmarco, Liberec, Czech Republic) at 22 °C, 25% humidity, −10/+60 kV, EMW speed 320 mm/s, rewinding speed 10 mm/min, distance of string and collector 160 mm. Prepared mats were stored at −80 °C until use.

### 4.7. Protein Content/Release Analysis of PVA_PL 

The protein release was analysed by sodium dodecyl sulfate–polyacrylamide gel electrophoresis (SDS-PAGE) after incubation of PVA mats containing proteins in phosphate-buffered saline (PBS, pH 7.4). Cumulative analysis was performed placing 10 mg of materials into 1 mL of PBS; subsequently, 200 µL of solution was taken for analysis at certain time intervals, and 200 µL of fresh PBS was added to keep the volume of 1 mL. Proteins were analysed after incubation at 37 °C for 1, 2, 4, 6, 8, 24, 72, and 168 h. Solutions after protein release (sample preparation described in [45] were analysed by 10% SDS-PAGE (1.5 h/90 V); proteins were visualised using the Coomassie Brilliant blue gel staining method.

### 4.8. PVA Crystallinity Analysis

The crystallinity of PVA was evaluated from thermal analysis, i.e., differential scanning calorimetry (DSC), of the prepared nanofibrous mats. Ten milligrams of samples was analysed using a differential scanning calorimeter DSC1/700 (Mettler Toledo, LLC, Columbus, OH, USA). Aluminium pots with samples were cooled to 0 °C and then heated to 300 °C at a rate of 10 °C/min. Heating was performed under inert conditions (nitrogen), and crystallinity was calculated from the recorded DSC curves. 

In addition, the crystallinity of the nanofibrous PVA materials was measured by X-ray diffraction (XRD) on a Bruker D8 Advance diffractometer equipped with a LynxeyePSD detector (Bruker, Billerica, MA, USA) and Cu K_α_1.2 radiation (40 kV and 40 mA), 0.02 mm Ni K_β_ absorber, 5–50° 2θ range and a 0.02° step scan with a sample rotation speed of 30 RPM. The relative proportion of crystalline regions in the samples was calculated using the following formula:

$$\alpha \;\left(\%\right)=\frac{I_{c}}{I_{c}+I_{a}}\cdot 100$$

where *α* is the degree of crystallinity, *I_c_* is the sum of the intensities below the crystalline peaks, and *I_a_* is the sum of the intensities below the amorphous sections of the spectra.

### 4.9. Cell Isolation, Cultivation and Characterisation

Lipoaspirates were harvested by liposuction from subcutaneous fat of tight regions of human female donors using a negative pressure of −700 mmHg. The procedure was accomplished in compliance with the tenets of the Declaration of Helsinki and under ethical approval issued by the Ethics Committee of the Bulovka Hospital in Prague (28 August 2014; 11 June 2019) and under Informed Consent of the patients. Adipose tissue-derived stem cells (ASCs) were isolated using the enzymatic digestion method first described by Estes and colleagues [78] and further modified by our group [79]. Briefly, after washing the lipoaspirate with PBS (Sigma-Aldrich, St. Louis, MO, USA) several times, an enzymatic digestion followed using PBS containing 1% (wt/vol) of bovine serum albumin (BSA; Sigma-Aldrich, St. Louis, MO, USA) and type I collagenase 0.1% (wt/vol) (Worthington, Lakewood, NJ, USA) for 1 h at 37 °C. After the centrifugation, the stromal vascular fraction (SVF) was obtained at the bottom of the test tubes. The SVF was washed, filtered through membranes with pores of 100 μm in diameter (Cell Strainer, BD Falcon, Corning, New York, NY, USA), and seeded into culture flasks (75 cm^2^, TPP, Trasadingen, Switzerland) in a density of 0.16 mL of original lipoaspirate/cm^2^. The ASCs were cultured in Dulbecco’s Modified Eagle Medium (DMEM) supplemented with 10% (vol/vol) of fetal bovine serum (FS, Cat. No. 10270-106 GIBCO, Waltham, MA, USA), gentamicin (40 µg/mL, Lek Pharmaceuticals d.d., Ljubljana, Slovenia) and human recombinant basic fibroblast growth factor (FGF2; 10 ng/mL; GenScript, Rijswijk, The Netherlands). The cells were passaged, when they reached 70–80% subconfluence. Flow cytometry analysis (Accuri C6 Flow Cytometer, BD Biosciences, San José, CA, USA) revealed that the ASCs were positive for cell surface antigens CD73 (ecto-50-nucleotidase, CD90 (immunoglobulin Thy-1), CD105 (endoglin) and CD29 (fibronectin receptor), all >95%, while they were almost negative for CD31 (platelet-endothelial cell adhesion molecule-1), CD34 (antigen of hematopoietic progenitor cells), CD45 (protein tyrosine phosphatase receptor type C), and CD146 (melanoma cell adhesion molecule or receptor for laminin, which is also considered to be a marker of pericytes, all <5%). For experiments, the ASCs were seeded at the passages 4–5. Human umbilical vein endothelial cells (ECs) were purchased from LONZA (Basel, Switzerland) and were seeded at the passages 3–4. 

### 4.10. Preparation of Collagen Hydrogels with Cells, Cell Cultivation

The lyophilised COL I was dissolved in 0.02 M acetic acid in the concentrations of 6 mg/mL and was stored at 4 °C for 5 days. The COL suspension was homogenised by means of a disintegrator (10,000 rpm, 10 min). Before collagen gel preparation, 32 mg of collagen particles per 1 mL of the gel were added into each well of a 24-well glass bottom plate (Cellvis, City, CA, USA, P24-1.5H-N). The hydrogel was prepared by mixing the COL suspension with the ASCs suspension in DMEM (Cat. No. 52100-021, Gibco Waltham, MA, USA), supplemented with 2% of FS (Cat. No. 10270-106 GIBCO), gentamicin (40 µg/mL, Lek Pharmaceuticals d.d., Ljubljana, Slovenia), 2-phospho-L-ascorbic acid trisodium salt (50 µg/mL, Cat. No. 49752, Sigma-Aldrich, St. Louis, MO, USA) and with sodium bicarbonate (7.5 wt.%). The ratio of COL, cell suspension and sodium bicarbonate solution was 1.766:1:0.0294 (*v/v/v*), respectively. Collagen suspension with the medium with cells was left to polymerise for at least 10–15 min at 37 °C, in a humidified atmosphere with 5% CO_2_, to reach pH around 7.4, and then 1.5 mL of cell culture medium was added above the gel. The change in pH from acidic to neutral and 37 °C caused the COL to polymerise into a hydrogel. Final COL concentration in gels was 3.3 mg/mL, and the final ASCs density was 500,000 cells/mL. 

The ASCs incorporated in the COL hydrogel without COL particles and in COL hydrogel reinforced with COL particles (part) were then cultured by the following manners: 

(1) In DMEM medium supplemented with 2% of FS, 40 µg/mL of gentamicin and 50 µg/mL of ascorbic acid (Cat. No. 49752, Sigma-Aldrich, St. Louis, MO, USA), mentioned above (samples labelled as DMEM_part);

(2) In the same medium with the addition of pure PVA nanomats (2 × 1 cm^2^; samples labelled as PVA_part);

(3) In the same medium with addition of PVA nanomats loaded with PL (2 × 1 cm^2^) representing an external drug delivery system (samples labelled as PVA_PL_part), and

(4) In the same medium supplemented with transforming growth factor β1 (TGF-β1, Cat. No. ab50036, Abcam, Cambridge, UK) and bone morphogenetic protein 4 (BMP-4, Cat. No. SRP6156-10UG, Sigma-Aldrich, St. Louis, MO, USA) at the concentration of 2.5 ng/mL, representing a culture medium proven for cell differentiation towards SMCs (samples labelled as TGF + BMP_part).

**Figure 12:**
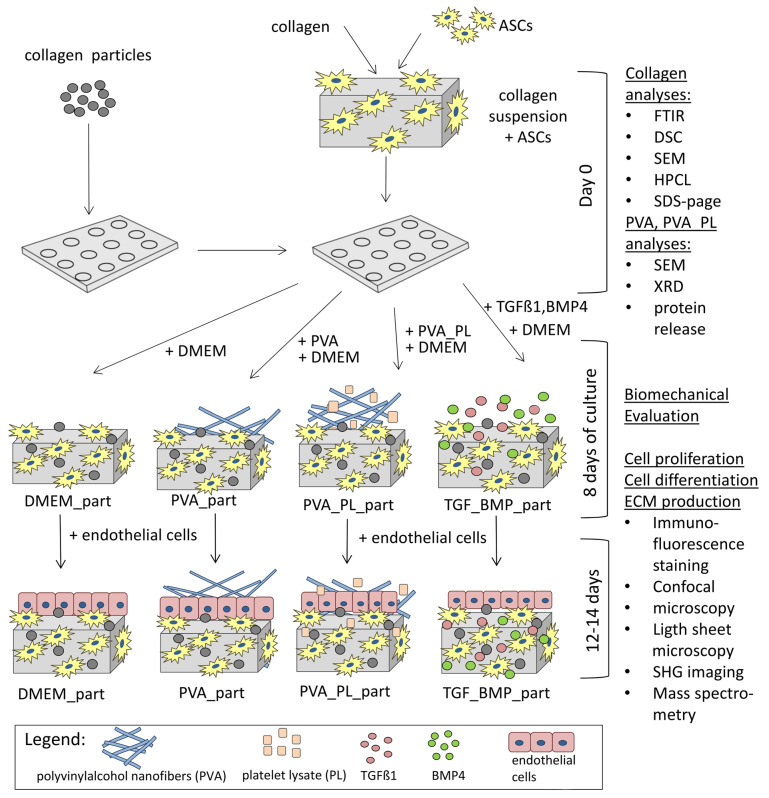
Scheme of the experiments.

In all four groups of samples, the medium was replaced twice per week during a 14-day culture. For endothelialisation of the gels reinforced with particles, ECs were seeded on the top of all four groups of samples at the density of 100,000 cells/well on day 8 after ASC incorporation, and cultured either in a pure EGM-2 medium (Promocell, Heidelberg, Germany) or in an EGM-2 medium with PVA nanomats, with PVA_PL namomats or with TGF + BMP until day 12 (for mass spectrometry) or until day 14 (for immunocytochemical staining). For measurement of biomechanical properties, the prepared gels with collagen particles were as thick as 1 cm, and the samples with ASCs cells were cultured for 5 days or were analysed immediately after preparation (day 0). The samples were incubated in a humidified atmosphere with 5% of CO_2_ at 37 °C. The scheme of the whole study is in Figure 12.

### 4.11. Visualisation of Living Cells

The ASCs cells cultured in TPP flasks were incubated with CellTracker^TM^ Red CMTPX dye or with CellTracker^TM^ Green CMFDA (both 25 µM, Thermo Fisher Scientific, Waltham, MA, USA) in DMEM with 10% of FS for 45 min. After washing with PBS, the cells were kept in a standard cell culture medium until the stained cells’ suspension was used for collagen gel preparation. The gels with embedded cells were vizualised using Andor Dragonfly 503—a spinning disk confocal microscope on day 14 of culture. The 3D and 2D projections were created, and cell densities were calculated via IMARIS software. 

### 4.12. Immunofluorescence Staining of Cell Differentiation Markers and ECM Components

The samples were fixed with 4% paraformaldehyde in PBS for 15 min at room temperature (RT), washed twice with PBS, and permeabilised with 1% BSA in PBS containing 0.1% Triton X-100 for 20 min. The samples were then washed with PBS, treated with 1% Tween for 20 min at RT and washed again with PBS.

The samples were incubated with a rabbit anti-calponin antibody (Abcam, Cat. No. ab46794 (EP798Y), dilution 1:200) at 4 °C overnight; then, they were washed twice with PBS and incubated with a mouse anti-alpha smooth muscle actin antibody (Sigma-Aldrich, St. Louis, MO, USA, clone 1A4, Cat No. A2547, dilution 1:200) at RT for 3.5 h. Then, the samples were washed twice with PBS and were incubated in a goat anti-rabbit secondary antibody conjugated with Alexa Fluor 488 (Thermo Fisher Scientific A11070, 1:400) for 90 min, washed with PBS and incubated in a goat anti-mouse secondary antibody conjugated with Alexa Fluor 546 (Thermo Fisher Scientific, A11003, 1:400) for 90 min. Finally, the samples were washed twice with PBS. 

For immunocytochemical staining of the co-culture of ASCs and ECs, mouse anti-alpha smooth muscle actin antibody (Sigma-Aldrich, St. Louis, MO, USA, clone 1A4, Cat No. A2547, dilution 1:200) was applied at 4 °C overnight, washed twice with PBS, and then rabbit anti-von Willebrand factor antibody (Sigma-Aldrich, St. Louis, MO, USA, F3520, dilution 1:200) for 3 h were applied. After washing twice with PBS, the anti-mouse and anti-rabbit secondary antibodies, mentioned above, were then applied sequentially at RT for 120 min each. The cell nuclei dye DAPI was added into the last secondary antibody solution (1 µg/mL). 

Similarly, immunofluorescence staining of fibronectin and type I collagen in reinforced collagen gels with ASCs on day 14 was performed using the primary antibodies as follows: the mouse monoclonal anti-human fibronectin antibody, clone IST-3 (Sigma-Aldrich, St. Louis, MO, USA, F079, 1:200), applied at RT for 3 h, and the rabbit polyclonal anti-type I collagen antibody (COSMO BIO CO., LTD, LSL-LB-1197, 1:200), applied at RT for 90 min. The secondary antibodies were F(ab’)2-goat anti-mouse IgG (H + L) cross-adsorbed secondary antibody, Alexa Fluor^TM^ 488 (Thermo Fisher Scientific, Waltham, MA, USA, A-11017, 1:400), which was applied at RT for 90 min and a mixture of AlexaFluor546 goat anti-rabbit IgG, cross-adsorbed secondary antibody (Thermo Fisher Scientific, Waltham, MA, USA, A11010, 1:400) and DAPI (1 µg/mL), applied at RT for 90 min. Quantification of the fluorescence signal of von Willebrand factor, fibronectin and type I collagen was performed by ImageJ software, and the data were normalised to the cell nuclei signal. 

### 4.13. Confocal Microscopy 

Images were obtained by the Dragonfly 503 spinning disk confocal microscope using software Fusion version 2.1.0.80 (Andor, Oxford Instruments, Tubney Woods, Abingdon, UK), equipped with objectives HC PL APO 10×/0.40 DRY and camera Zyla 4.2 PLUS sCMOS—2048 × 2048 pixels. Images were acquired with a voxel edge size of 6.5 µm and a z-step of 5 µm using a 25 μm pinhole size. Cells stained by CellTracker Red CMTPX or CellTracker Green CMFDA (Thermo Fisher Scientific, Waltham, MA, USA, 25 µM) were excited by a 561 nm laser; emissions were collected in a range of 575–625 nm for the red tracker, excited by a 488 nm laser, and collected in a range of 500–550 nm for the green tracker.

### 4.14. Lightsheet Microscopy

Lightsheet microscopy 3D images were acquired with a Zeiss Z.1 lightsheet microscope using the 10×/0.2 NA excitation and 20×/1.0 water immersion detection objective lenses, 488 nm and 561 nm excitation and the respective green (505–545 nm) and red (575–615 nm) emission. Image processing and visualisation were performed in ZEN (Zeiss, GmbH, Aalen, Germany) and Arivis Vision 4D (Zeiss, GmbH, Aalen, Germany) software, respectively.

### 4.15. Nonlinear Imaging—Second Harmonic Generation

Second Harmonic Generation (SHG), able to visualize mature collagen fibers [28], was performed on a Zeiss LSM 780 microscope system (Zeiss, GmbH, Aalen, Germany), comprised of an Axio Observer inverted microscope stand, LSM 780 laser scanner head and Coherent Chameleon tunable femtosecond laser. For SHG, 860 nm excitation was used, together with 430/15 detection using the internal LSM 780 spectral detector. The sample was kept at 10 °C with the help of a cooler (Okolab, S.r.l., Pozzuoli, Italy).

### 4.16. Bruker Microscopy

Samples were measured using multiphoton microscope Bruker Ultima (Bruker Corporation, Billerica, MA, USA) using the CFI75 Apo 25XC W 1300 objective (1.1 NA). Images of samples were captured as 12-bit images of different z-planes 2 µm apart at 1024 × 1024 pixel resolution with 525.45 × 525.45 µm using Prairie View (Bruker Corporation, Billerica, MA, USA). The excitation was carried out with two different wavelengths, 940 nm and 1100 nm (or 760 nm). The emission signal was filtered through bandpass filters ET525/70m-2P and ET595/50m-2P (or 450/50m-2P) (Chroma Technology Corp., Bellows Falls, VT, USA) and detected with GaAsP photosensors sensitive in the visible light region (Hamamatsu, Hamamatsu, Japan). Three-dimensional projections were created using ImageJ and IMARIS software (Oxford Instruments, Tubney Woods, Abingdon, UK).

The two-channel lightsheet data were processed in Arivis Vision 4D version 3.1.3. After stitching, data were denoised with a 2D median filter, and background correction was applied. Objects were segmented using the global intensity threshold, and their morphological and intensity features were exported for further statistical analysis.

### 4.17. Evaluation of Biomechanical Properties

Biomechanical testing of the collagen hydrogel was performed using an unconfined compression test. The MTS Mini Bionix 858.02 testing system (MTS, Eden Prairie, MN, USA) equipped with 10 N force sensor (sensitivity 0.001 N) was used for the compression tests in quasi-static mode at a constant cross head loading speed of 2.0 mm/min. A total of 5 cycles were realised, i.e., 5 times loaded to 1 N and 5 times unloaded to 0.05 N. The hydrogel disks for this test were placed between two stainless steel cylinders at room temperature (24 °C). The force and the deformation data were recalculated into the stress–stain curves. The mechanical properties of the composite material were characterised by the initial Young modulus of elasticity, total deformation energy and the ratio of elastic energy, i.e., the ratio between the elastic deformation energy and the total deformation energy. The data were acquired by the MTS Mini Bionix testing system and were analysed in Matlab 2020b (MathWorks, Natick, MA, USA). The environmental conditions were recorded by a COMET (Comet System, S.R.O., Roznov pod Radhostem, Czech Republic) digital thermometer hygrometer.

### 4.18. Mass Spectrometry—Label Free Quantification (MS LFQ)

Biological samples (n = 4 in each group) were harvested, and MS LFQ analysis was performed. Briefly, each sample was lyophilisated and 10 mg of dry weight was taken for analyses. Samples were homogenised in liquid nitrogen, and 0.3 mL of 0.1 M ammonium bicarbonate (pH = 7.8) was added. After 30 min of sonication and centrifugation (10,000× *g*; 5 min), the supernatant was extracted and used for subsequent analysis (both pellet and supernatant were stored at −25 °C). Supernatant samples were heated at 105 °C for 5 min., digested with trypsin and desalted on Empore C18 columns, dried in Speedvac (Thermo Fisher Scientific, Waltham, MA, USA). Samples were dissolved in 0.1% TFA + 2% acetonitrile. About 0.5 µg of peptide digests were separated on 50 cm C18 column using 2.5 h elution gradient and analysed in a DDA mode on Orbitrap Exploris 480 mass spectrometer (Thermo Fisher Scientific, Waltham, MA, USA). The resulting raw files were processed in MaxQuant (v. 1.6.17.0) with label-free quantification (LFQ) algorithm MaxLFQ. The search was performed at 0.01 false discovery rate (FDR) levels using FASTA files from UniProt (release 2021_01). Downstream analysis and visualisation were performed in Perseus (v. 1.6.15.0.). The *p*-value returned was corrected by FDR based on a frequency histogram. An FDR adjusted *p*-value threshold was less than 0.01.

### 4.19. Statistical Analysis

For cell experiments, an all pairwise multiple comparison procedure (ANOVA, Student–Newman–Keuls Method) was used. Biomechanical data were analysed using a Student’s *t*-test, and statistical significance was considered for *p* value ≤ 0.05. The statistical analysis of mass spectrometry is described in the previous subsection.

## 5. Conclusions

Collagen gels reinforced with collagen particles, incorporated with ASCs and endothelialised with HUVECs were prepared and cultured either in a medium with TGF-β1 and BMP-4 (TGF + BMP_part sample), or in a medium with a newly developed external drug delivery system, represented by a nanofibrous PVA mesh releasing platelet lysate (PVA_PL_part sample). Cell-material constructs incubated only in a standard cell culture medium (DMEM_part) or in this medium with pure PVA nanomats (PVA_part) served as negative controls. 

Gel reinforcement with collagen particles prevented the planar shrinkage of all gels until day 7. On day 14, significant gel shrinkage was observed with TGF + BMP_part gels either with or without endothelial cells. On the contrary, the minimum shrinkage was observed with PVA_PL_part gel regardless of its endothelialisation. 

Endothelial cells formed a confluent layer on all gels; however, TGF + BMP_part gels evinced less homogeneous coverage with endothelial cells. 

The differentiation of ASCs towards SMCs, assessed by the intensity of calponin immunofluorescence staining as well as proteomic analysis, was strongest in TGF + BMP_part samples, moderate in PVA_PL_part and PVA_part samples, and the lowest in DMEM_part samples.

The highest concentrations of proteins involved in ECM synthesis and remodelling were found in TGF + BMP_part samples, lower in PVA_PL_part and PVA_part samples, and the lowest in DMEM_part samples.

In all samples, we observed improved mechanical properties on day 5 after seeding compared to day 0. The PVA_PL samples seem to have a slightly higher ratio of elastic energy, and TGF + BMP_part a slightly higher total deformation energy value.

PVA_PL_part samples with an external drug delivery system, releasing platelet bioactive components into the culture medium, represent a more natural way of stimulating ASCs proliferation, differentiation, ECM production and remodelling than the direct addition of TGF-β1 and BMP-4 into the medium. The slower effect of the external PVA_PL system, probably limited by the amount of released PL components, was balanced with homogeneous endothelialisation and minimum gel shrinkage. 

## Figures and Tables

**Table 1:** Composition of COL lyophilizate isolated from porcine skin. “*” marked lower and upper quartile.

Amino Acid	Number of AA Residues/1000 Units	Amino Acid	Number of AA Residues/1000 Units
Asp + Asn	48	Cys	2
Glu + Gln	76	Met	6
Thr	17	Tyr	3
Ser	32	Phe	15
Gly	319	Lys	31
Ala	111	His	15
Val	31	Arg	52
Ile	11	Pro	121
Leu	27	Hyp	81
Protein (wt%)		58.45	
Hyp (wt%)		5.61 (5.28–5.82) *	
Degree of hydroxylation (%)		40	
GAGs (wt%)		3.92 (3.52–4.81) *	
Lipids (wt%)		25.49 (22.98–32.82) *	
Water (%)		7.23 (7.05–7.35) *	

**Table 2:** Proteins involved in cell differentiation produced by ASCs and ECs in DMEM_part, PVA_part, PVA_PL_part and TGF + BMP_part samples during a 12-day culture; ECs were seeded on day 8 after ASCs seeding. Data are expressed as a multiple of the value of control group (12-day culture/DMEM on day 0). In all cases except for those marked, “n.s. D0” is declared as significantly increased protein concentration, FDR adjusted *p* value ≤ 0.01, “n“ refers to the average number of specific protein peptides detected in the sample groups. The bold numbers and characters in brackets (a; b; c; d) mean the significant differences among 12-day culture samples (DMEM_part = “a“; PVA_part = “b“; PVA_PL_part = “c“; TGF + BMP_part = “d“). The values above 3 are coloured in light brown, and significant changes are marked in bold.

Differentiation	DMEM_Part (a)	PVA_Part (b)	PVA_PL_Part (c)	TGF + BMP_Part (d)
Alpha-adducin (ADD1)	3.37 *n* = 2	**2.62 (d)** ***n* = 1.25**	1.89 n.s. D0 *n* = 1	1.66 n.s. D0 *n* = 1.5
Alpha-parvin (PARVA)	3.39 *n* = 3.75	3.82 *n* = 3	3.29 *n* = 3.25	2.88 *n* = 3.5
Caldesmon 1 (CALD1)	1.77 *n* = 8.25	1.05 n.s. D0 *n* = 6	**1.04** n.s. D0 **(b), *n* = 6.75**	**2.62** n.s. D0 **(b), *n* = 5.75**
Calponin-2 (CNN2)	3.11 *n* = 2	3.47 *n* = 2	3.25 *n* = 1.75	3.75 *n* = 2
Cofilin-2 (CFL2)	1.99 *n* = 2.75	1.86 *n* = 2.75	1.60 *n* = 2.75	1.62 *n* = 2.75
Endothelial differentiation-related factor 1 (EDF1)	2.13 n.s. D0 *n* = 1.5	2.66 *n* = 1.5	2.71 *n* = 1.75	2.97 *n* = 2
Endothelial monocyte-activating polypeptide 2 (AIMP1)	3.72 *n* = 1	3.62 *n* = 0	3.55 *n* = 0.75	3.57 *n* = 1
F-actin-capping protein subunit alpha-1 (CAPZA1)	3.51 *n* = 7	3.24 *n* = 6.25	3.36 *n* = 6.5	3.14 *n* = 7
Fascin (FSCN1)	4.51 *n* = 3.75	**4.82 (d)** ***n* = 3.75**	4.40 *n* = 4	3.81 *n* = 4
Filamin-A (FLNA)	6.37 *n* = 39	6.16 *n* = 36.25	5.89 *n* = 36	6.43 *n* = 39.75
Filamin-C (FLNC)	4.91 *n* = 25	4.91 *n* = 23	5.01 *n* = 25	5.60 *n* = 27.5
LIM and SH3 domain protein 1 (LASP1)	3.70 *n* = 3.5	3.18 *n* = 3.5	3.11 n.s. D0 *n* = 3.75	3.13 *n* = 4
Myosin light polypeptide 6 (MYL6)	3.50 *n* = 3.75	3.62 *n* = 4.25	3.26 *n* = 4	3.62 *n* = 4.5
Myosin phosphatase Rho-interacting protein (MPRIP)	1.43 n.s. D0 *n* = 0.5	1.13 n.s. D0 *n* = 0	1.76 n.s. D0 *n* = 0.25	**2.72 (b, c)** ***n* = 1.5**
Nexilin (NEXN)	1.98 n.s. D0 *n* = 1	**1.47 (d)** ***n* = 1**	1.41 n.s. D0 *n* = 0.75	1.38 n.s. D0 *n* = 1
Platelet endothelial cell adhesion molecule (PECAM1)	1.85 n.s. D0 *n* = 0.75	1.79 n.s. D0 *n* = 1	1.29 n.s. D0 *n* = 0.75	1.33 *n* = 1
Plectin (PLEC)	4.95 *n* = 48.5	4.92 *n* = 46.25	4.96 *n* = 45.75	4.95 *n* = 45.25
Transgelin (TAGLN)	1.59 *n* = 3.75	1.35 n.s. D0 *n* = 3	1.38 n.s. D0 *n* = 4	**3.35 (b, c)** ***n* = 4**
Tropomodulin-3 (TMOD3)	4.18 *n* = 4	3.89 *n* = 2.25	3.92 *n* = 2.75	4.23 *n* = 3.25
Tropomyosin alpha-1 chain (TPM1)	1.11 n.s. D0 *n* = 4.75	1.23 n.s. D0 *n* = 5	1.62 n.s. D0 *n* = 4.5	**2.05 (a, b, c)** ***n* = 6.75**
Tropomyosin alpha-3 chain (TPM3)	4.01 *n* = 4.5	4.14 *n* = 5.75	3.85 *n* = 5.5	3.61 *n* = 6.5
Tropomyosin alpha-4 chain (TPM4)	1.99 n.s. D0 *n* = 7.5	1.48 n.s. D0 *n* = 7.5	1.39 n.s. D0 *n* = 8	1.16 *n* = 8.5
Utrophin (UTRN)	2.075 *n* = 3.75	2.70 *n* = 3	1.95 *n* = 3.25	1.62 *n* = 3
Vinculin (VCL)	2.39 *n* = 20.75	2.39 *n* = 20.5	2.31 *n* = 21.25	2.27 *n* = 19.25
von Willebrand factor (VWF)	2.84 *n* = 8	3.46 *n* = 9	3.56 *n* = 8	3.31 *n* = 10

**Table 3:** Proteins of ECM produced by ASCs and ECs in DMEM_part, PVA_part, PVA_PL_part and TGF + BMP_part samples during a 12-day culture; ECs were seeded on day 8 following ASCs seeding. Data are expressed as a multiple of the value of control group (12-day culture/DMEM on day 0). In all cases except for those marked, “n.s. D0” is declared significantly increased protein concentration, FDR adjusted *p* value ≤ 0.01, “n“ refers to the average number of specific protein peptides detected in the sample groups. The bold numbers and characters in brackets (a; b; c; d) mean the significant differences among 12-day culture samples (DMEM_part = “a“; PVA_part = “b“; PVA_PL_part = “c“; TGF + BMP_part = “d“). The values above 3 are coloured in light brown, and significant changes are marked in bold.

Extracellular Matrix	DMEM_Part (a)	PVA_Part (b)	PVA_PL_Part (c)	TGF + BMP_Part (d)
Basement membrane-specific heparan sulfate proteoglycan core protein (HSPG2)	6.17 *n* = 13	6.02 *n* = 13.5	6.08 *n* = 13	5.49 *n* = 10.75
Collagen alpha-1(V) chain (COL5A1)	0.65 n.s. D0 *n* = 2.75	0.85 *n* = 3.5	0.8 *n* = 3.5	**1.10** n.s. D0 **(b, c) *n* = 4**
Collagen alpha-1(XII) chain (COL12A1)	0.16 *n* = 0.75	0.16 *n* = 0.5	0.15 *n* = 0.75	**0.63** n.s. D0 **(a, b, c)** ***n* = 4.5**
Collagen alpha-1(VII) chain (COL7A1)	0.65 n.s. D0 *n* = 0	0.92 n.s. D0 *n* = 0	0.7 n.s. D0 *n* = 0	**3.74 (a, b, c)** ***n* = 1.75**
Collagen triple helix repeat-containing protein 1 (CTHRC1)	1.36 n.s. D0 *n* = 0.75	1.34 *n* = 1	1.19 *n* = 1	2.13 (c) *n* = 1
Decorin (DCN)	4.47 *n* = 3.5	3.26 *n* = 3.25	3.66 *n* = 2.75	3.66 *n* = 1
Extracellular matrix protein 1 (ECM1)	2.81 *n* = 3.25	**3.86 (d)** ***n* = 4**	**3.05 (d)** ***n* = 3.25**	1.39 n.s. D0 *n* = 2.5
Fibrillin-1 (FBN1)	1.99 n.s. D0 *n* = 0.75	1.99 n.s. D0 *n* = 0.5	n.s. D0 *n* = 1	1.65 *n* = 0.75
Fibronectin (FN1)	6.09 *n* = 29.25	5.80 *n* = 27	5.77 *n* = 29	7.20 *n* = 36
Fibulin-1 (FBLN1)	2.68 *n* = 2.25	2.22 *n* = 2	2.32 *n* = 2	2.28 *n* = 1.75
Galectin-3-binding protein (LGALS3BP)	2.61 *n* = 2	**2.60 (d)** ***n* = 2**	**2.99 (d)** ***n* = 2**	1.54 n.s. D0 *n* = 1.75
Laminin subunit beta-1 (LAMB1)	5.05 *n* = 9.25	5.26 *n* = 10	5.29 *n* = 9.75	4.89 *n* = 9
Laminin subunit gamma-1 (LAMC1)	4.84 *n* = 11	4.85 *n* = 11	4.97 *n* = 11.25	4.43 *n* = 9.25
Laminin subunit alpha-2 (LAMA2)	1.47 n = 1.25	1.19 n.s. D0 n = 0.75	1.11 n.s. D0 n = 0.75	1.31 n = 1
Laminin subunit alpha-4 (LAMA4)	4.68 *n* = 7	**4.70 (d)** ***n* = 5.25**	**4.77 (d)** ***n* = 6.5**	2.53 *n* = 4
Lumican (LUM)	2.23 *n* = 1.5	2.36 *n* = 2	2.41 *n* = 2	2.59 *n* = 1
Nidogen-1 (NID1)	2.68 *n* = 3	**2.52 (d)** ***n* = 3.75**	**2.81 (d)** ***n* = 3**	1.24 n.s. D0 *n* = 2.5
Tenascin (TNC)	0.54 n.s. D0 *n* = 1.75	0.77 n.s. D0 *n* = 1.25	0.52 n.s. D0 *n* = 1.75	**3.28 (b, c)** ***n* = 0**
Thrombospondin-4 (THBS4)	3.45 *n* = 1.25	**4.36 (d)** ***n* = 1.25**	**4.12 (d)** ***n* = 1.25**	2.65 *n* = 1

**Table 4:** Proteins involved in extracellular matrix remodelling produced by ASCs and ECs in DMEM_part, PVA_part, PVA_PL_part and TGF + BMP_part samples during a 12-day culture; ECs were seeded on day 8 following ASCs seeding. Data are expressed as a multiple of the value of control group (12-day culture/DMEM on day 0). In all cases except for those marked, “n.s. D0” is declared significantly increased protein concentration, FDR adjusted *p* value ≤ 0.01, “n“ refers to the average number of specific protein peptides detected in the sample groups. The bold numbers and characters in brackets (a; b; c; d) mean the significant differences among 12-day culture samples (DMEM_part = “a“; PVA_part = “b“; PVA_PL_part = “c“; TGF + BMP_part = “d“). The values above 3 are coloured in light brown, significant changes are marked in bold.

REMODELLING	DMEM (a)	PVA (b)	PVA_PL (c)	TGF + BMP (d)
Collagenase, type IV (MMP2)	6.27 *n* = 3.5	6.27 *n* = 3.75	6.42 *n* = 3.75	6.30 *n* = 3.25
Disintegrin and metallo-proteinase with thrombo-spondin motifs 1 (ADAMTS1)	3.46 *n* = 1	3.46 *n* = 1	3.93 *n* = 1	2.86 *n* = 1
Interstitial collagenase, MMP1	3.88 *n* = 4.25	**5.04 (d)** ***n* = 6.5**	**4.97 (d)** ***n* = 5.75**	3.06 *n* = 3
Lysyl oxidase homolog 2 (LOXL2)	1.70 n.s. D0 *n* = 0.75	1.39 n.s. D0 *n* = 1	1.25 n.s. D0 *n* = 1	1.50 *n* = 1
Matrix metalloproteinase-14 (MMP14)	1.48 n.s. D0 *n* = 0.75	1.26 n.s. D0 *n* = 0.75	0.6 *n* = 1	0.6 *n* = 6.25
Matrix-remodelling-associated protein 7 (MXRA7)	1.56 n.s. D0 *n* = 1.25	1.95 n.s. D0 *n* = 1	1.79 n.s. D0 *n* = 1	**3.48 (b)** ***n* = 2**
Metalloproteinase inhibitor 1 (TIMP1)	5.81 *n* = 2	6.77 *n* = 2	6.47 *n* = 2	6.14 *n* = 2
Periostin (POSTN)	1.17 n.s. D0 *n* = 4.25	1.18 n.s. D0 *n* = 4.25	1.01 n.s. D0 *n* = 4	**1.96 (a, b, c)** ***n* = 10.5**
Procollagen C-endopeptidase enhancer 1 (PCOLCE)	3.04 *n* = 1	1.86 n.s. D0 *n* = 1	2.47 n.s. D0 *n* = 1	2.96 *n* = 1
Procollagen-lysine, 2-oxoglutarate 5-dioxygenase 1 (PLOD1)	2.37 n.s. D0 *n* = 2	2.68 *n* = 2	2.59 *n* = 2	2.45 *n* = 2
Procollagen-lysine, 2-oxo-glutarate 5-dioxygenase 2 (PLOD2)	2.91 *n* = 5.5	3.59 *n* = 5.75	3.19 *n* = 5.25	4.80 *n* = 7.25
TGF-beta-induced protein ig-h3 (TGFBI)	1.41 n.s. D0 *n* = 4	1.42 *n* = 6.25	1.32 *n* = 5	2.79 *n* = 7.25
Transforming growth factor beta-1-induced transcript 1 protein (TGFB1I1)	1.17 n.s. D0 *n* = 0.25	1.24 n.s. D0 *n* = 0	1.82 n.s. D0 *n* = 0	**1.97 (c)** ***n* = 0.75**

## Data Availability

The data presented in this study are available on request from the corresponding authors.

## Supplementary Materials

The following supporting information can be downloaded at: https://www.mdpi.com/article/10.3390/ijms24065692/s1.
