# Supplementary figures and images for: Adipose-Derived Stem Cells in Reinforced Collagen Gel: A Comparison between Two Approaches to Differentiation towards Smooth Muscle Cells
Source: Int J Mol Sci. 2023 Mar 16;24(6):5692. doi: 10.3390/ijms24065692 (PMC10058441; doi:10.3390/ijms24065692)

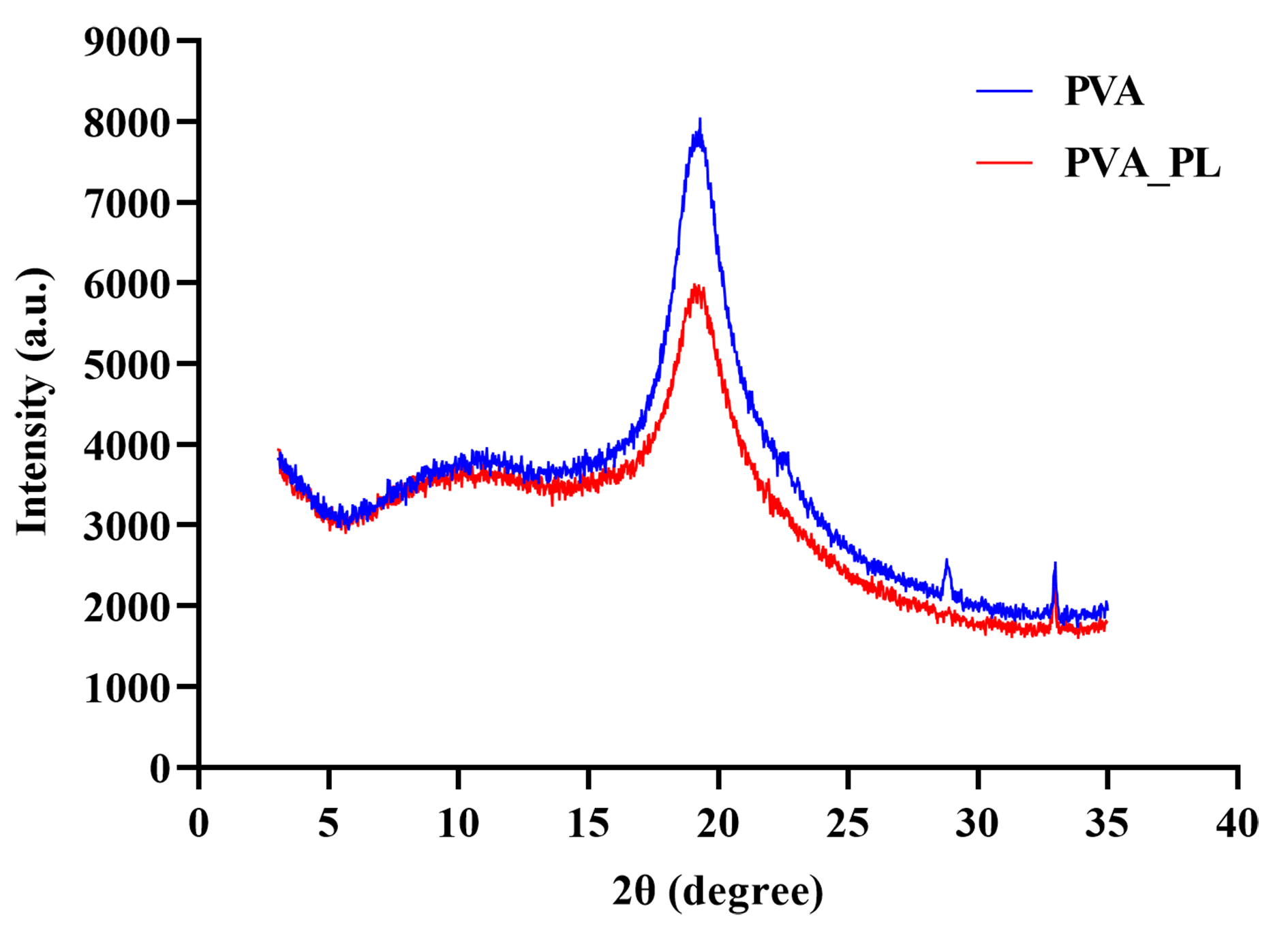

Supplement: Supplementary file 1 [file ijms-24-05692-s001.zip › ijms-2250373-supplementary/Supplementary files/Fig S1_XRDcm600dpi.tif]

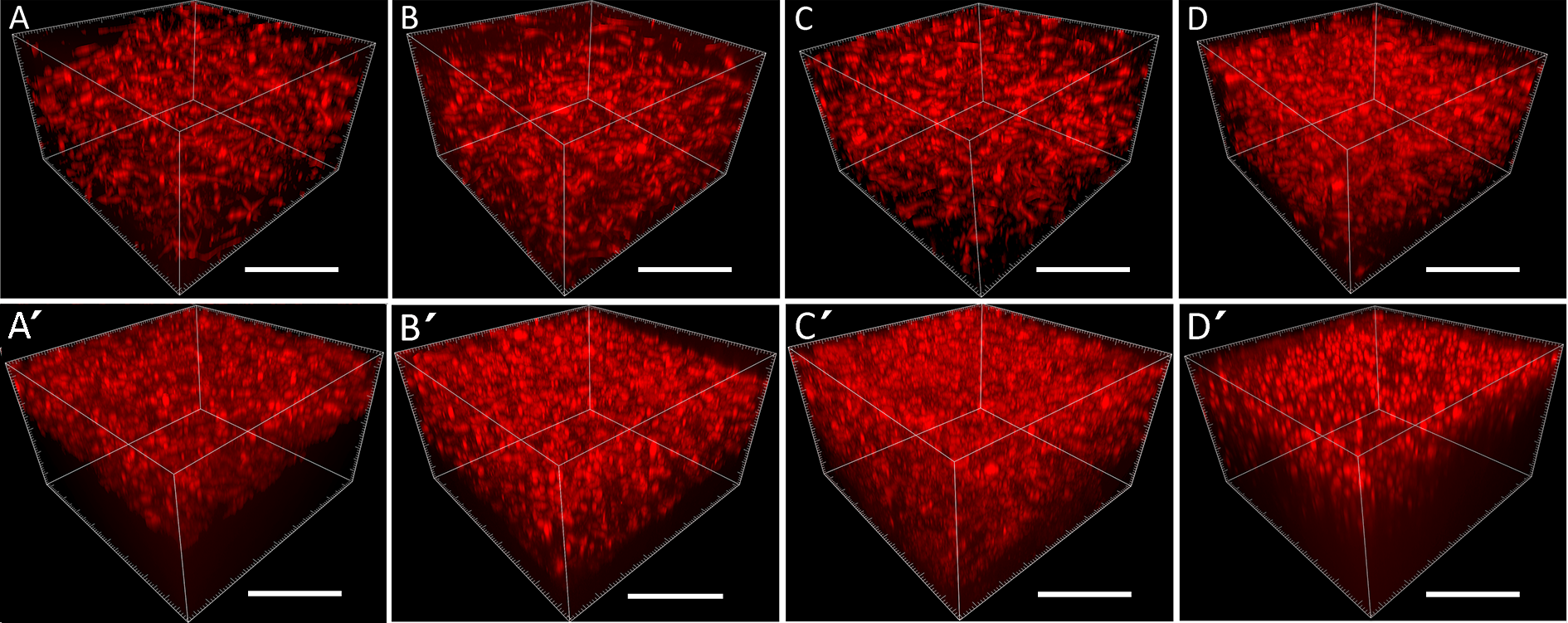

Supplement: Supplementary file 1 [file ijms-24-05692-s001.zip › ijms-2250373-supplementary/Supplementary files/Fig S2_red tracker D1_D6morphologyFlat4_300dpi.tif]

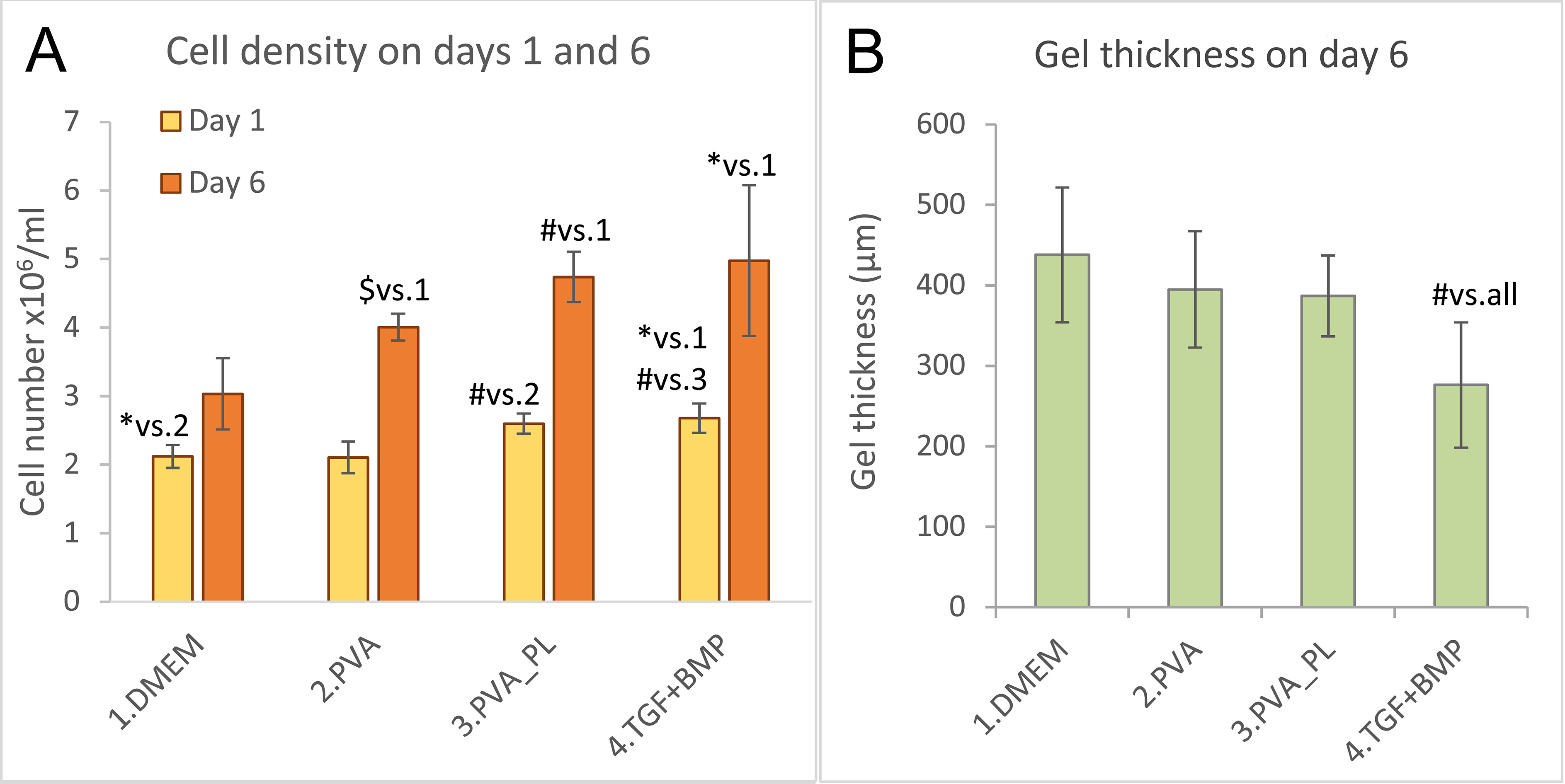

Supplement: Supplementary file 1 [file ijms-24-05692-s001.zip › ijms-2250373-supplementary/Supplementary files/Fig S3_cell number_shrink compFlatOrez3fLAT2_600di.tif]

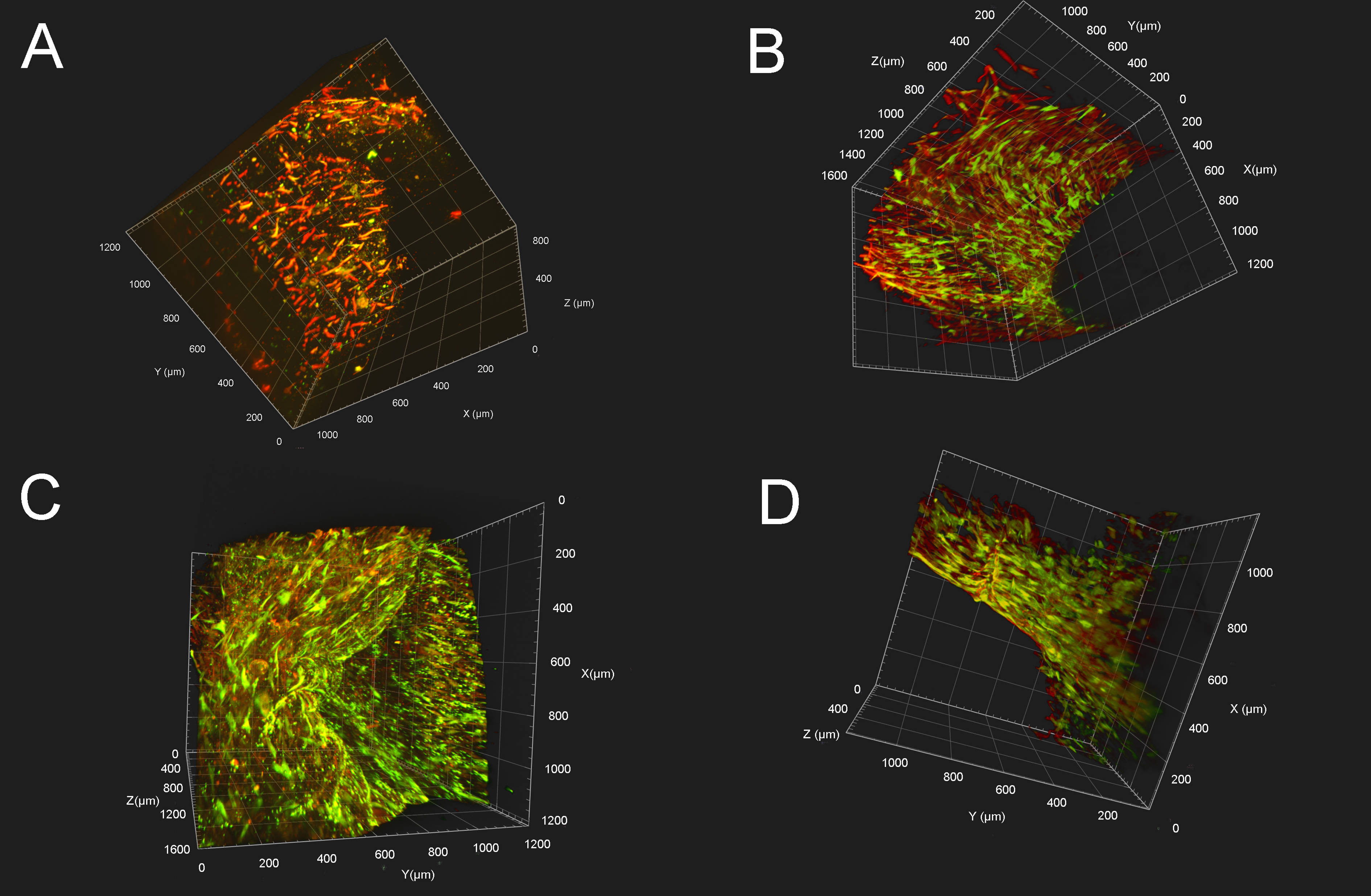

Supplement: Supplementary file 1 [file ijms-24-05692-s001.zip › ijms-2250373-supplementary/Supplementary files/Fig S4 6D_DMEM_PVA_PVAPL_TGFAktCalpComp600dpi16cm.tif]

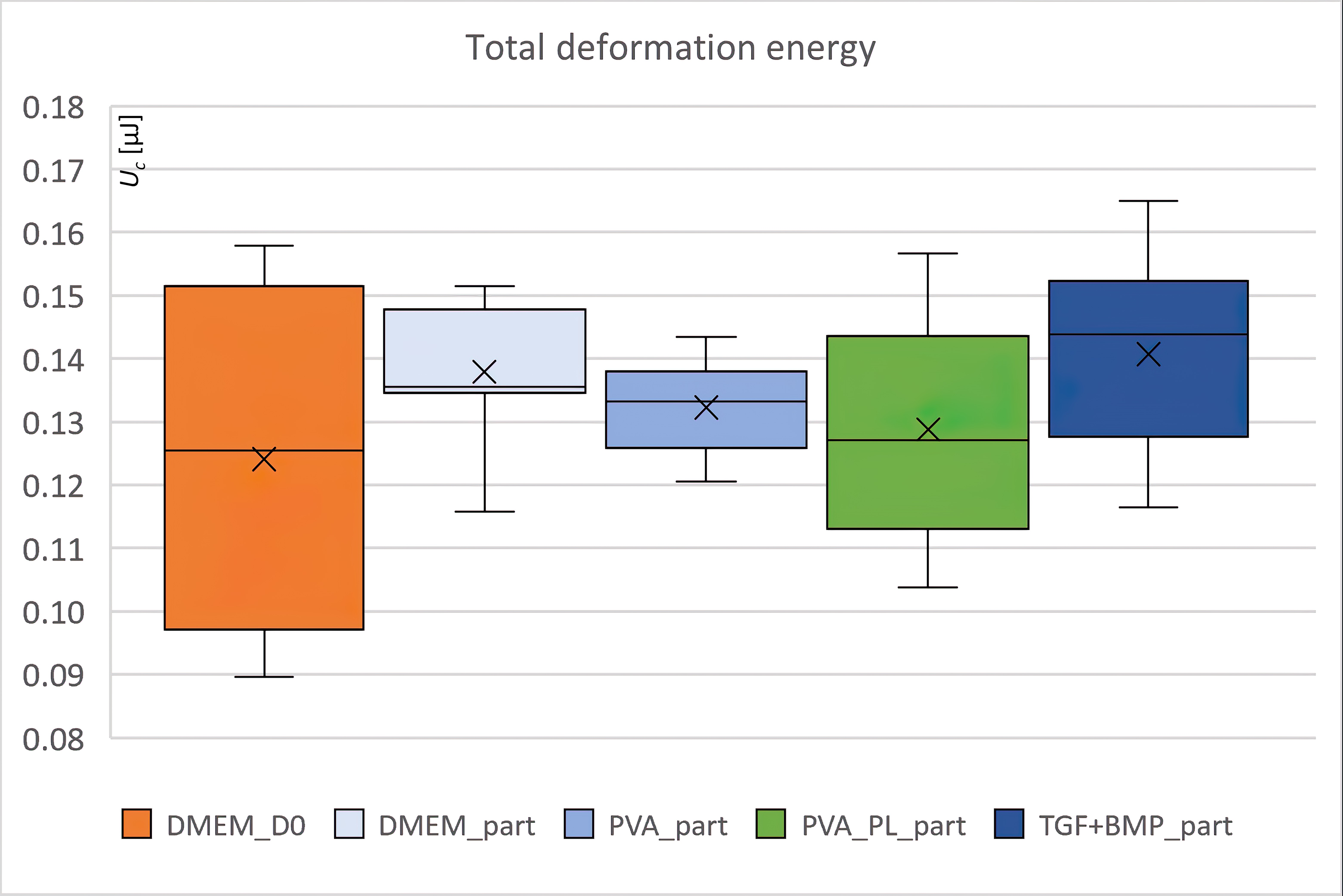

Supplement: Supplementary file 1 [file ijms-24-05692-s001.zip › ijms-2250373-supplementary/Supplementary files/Fig S5 total_deformation_energy600dpi.tif]

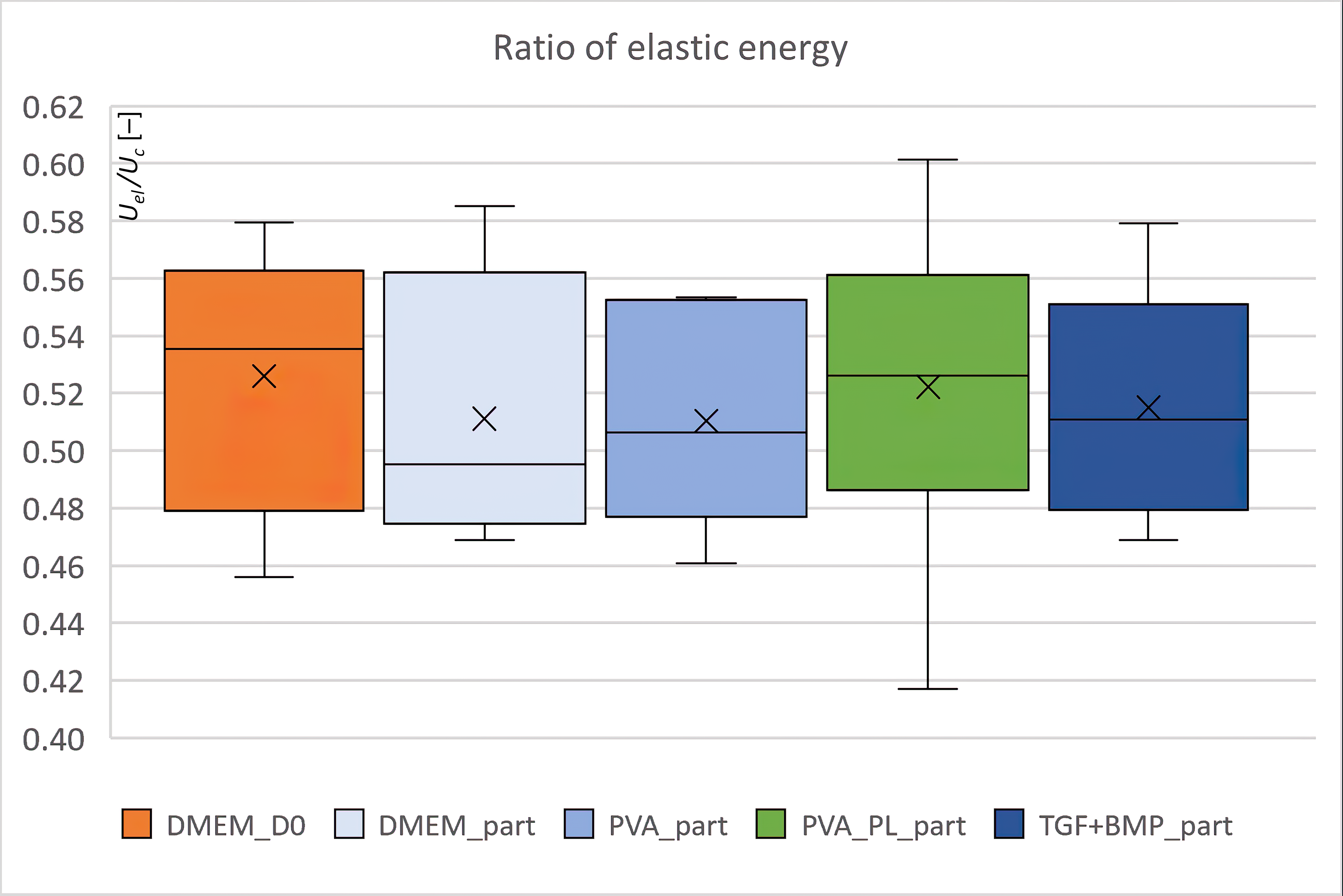

Supplement: Supplementary file 1 [file ijms-24-05692-s001.zip › ijms-2250373-supplementary/Supplementary files/Fig S6 ratio_of_elastic_energy600dpi.tif]
